# Modelling COVID-19 in the North American region with a metapopulation network and Kalman filter^☆^

**DOI:** 10.1016/j.epidem.2025.100818

**Published:** 2025-01-26

**Authors:** Matteo Perini, Teresa K. Yamana, Marta Galanti, Jiyeon Suh, Roselyn Kaondera-Shava, Jeffrey Shaman

**Affiliations:** a Department of Environmental Health Sciences, Mailman School of Public Health, Columbia University, 722 W 168th St, New York, NY 10032, United States; b Columbia Climate School, Columbia University, Level A Hogan, 2910 Broadway, New York, NY 10025, United Statess

**Keywords:** COVID-19, Metapopulation model, North America, Transmission dynamics, Bayesian inference

## Abstract

**Background:**

Understanding the dynamics of infectious disease spread and predicting clinical outcomes are critical for managing large-scale epidemics and pandemics, such as COVID-19. Effective modeling of disease transmission in interconnected populations helps inform public health responses and interventions across regions.

**Methods:**

We developed a novel metapopulation model for simulating respiratory virus transmission in the North America region, specifically for the 96 states, provinces, and territories of Canada, Mexico, and the United States. The model is informed by COVID-19 case data, which are assimilated using the Ensemble Adjustment Kalman filter (EAKF), a Bayesian inference algorithm. Additionally, commuting and mobility data are used to build and adjust the network and movement across locations on a daily basis.

**Results:**

This model-inference system provides estimates of transmission dynamics, infection rates, and ascertainment rates for each of the 96 locations from January 2020 to March 2021. The results highlight differences in disease dynamics and ascertainment among the three countries.

**Conclusions:**

The metapopulation structure enables rapid simulation at a large scale, and the data assimilation method makes the system responsive to changes in system dynamics. This model can serve as a versatile platform for modeling other infectious diseases across the North American region.

## 1. Introduction

Mathematical models have been used to simulate infectious diseases outcomes, infer transmission dynamics, and predict future disease burden. These tools can inform public health strategies by testing control methods and identifying effective interventions (Pagel and Yates, 2022). During the SARS-CoV-2 pandemic, unprecedented data availability enabled application of mathematical models in many locations and at different geographical scales worldwide (Zhou et al., 2023; Zhang et al., 2022; Pei et al., 2020; Li et al., 2020). Metapopulation modeling approaches provide an efficient framework for simulating and evaluating the spatiotemporal progression of infectious disease over large geographic areas. In this model form, populations are aggregated within typically fixed geographic units (e.g. provinces, cities), which allows resolution of spatial disease patterns without the computational expense and micro-behavioral assumptions required for agent-based models. At large geographical scales, agent-based models require high-performance computing (HPC) clusters. For example, Bhattacharya et al. developed a platform to enable the real-time execution of an agent-based COVID-19 model for the United States on more than ten thousand CPU cores (Bhattacharya et al., 2024).

Many metapopulation models have been developed to describe the dynamics of infectious diseases at different geographic scales (Kong et al., 2022; Wang et al., 2022); however, a key challenge in model development lies in accurately determining the movement patterns of individuals among subpopulations. Some metapopulation models use fixed or arbitrary sized geographical areas (cells) as subpopulations, which can then be aggregated to match the resolution of available case, census and movement data (Venkatramanan et al., 2021; Balcan et al., 2010). One example of a multi-national system is the GLEAM (Global Epidemic And Mobility) platform (Balcan et al., 2010; Pastore-Piontti et al., 2016; Zhang et al., 2017), which estimates the flux of individuals among arbitrary subpopulations centered around major transportation hubs (usually airports) and uses commuting and air travel data (Chinazzi et al., 2020).

In 2020, the US reported the highest number of COVID-19 cases and deaths globally, with the first case identified in Washington state on January 20th and three pandemic waves manifesting during the year (Pei et al., 2021). Canada reported its first case in Toronto on January 25th and experienced a decline in cases during the summer followed by a resurgence in the fall. Mexico had a similar epidemiological history, but the first wave developed later during the summer (CSSEGISandData, 2024). The literature currently lacks a comprehensive COVID-19 model for the North American region at continental scale. Here, we present a metapopulation susceptible-exposed-infectious-recovered (SEIR) for COVID-19 for the majority of the North American region (i.e. Canada, United States and Mexico) from the beginning of the pandemic to widespread availability and distribution COVID-19 vaccines. The model is coupled with a data assimilation algorithm and is informed by daily COVID-19 cases and a commuting network that is adjusted by daily mobility trends. In this model the flux of individuals among subpopulations of the metapopulation model is calculated using the daily work commuting patterns coupled with random movements among the geographical locations, as in previous work (Pei et al., 2020).

The model developed here provides temporal estimates of transmission and ascertainment rates for COVID-19 for the North American Region, filling a gap in the existing literature regarding models for this region. The flux patterns and the continental-level metapopulation structure used in this study helps investigation of disease dynamics of COVID-19 at a larger scale and can reveal dynamics that are not discernible at smaller scale. Lastly, the work commuting matrix structure developed for this study can be paired to different compartmental models and disease surveillance data to study the dynamics of other infectious diseases such as influenza.

## 2. Methods

**Fig. 1.**
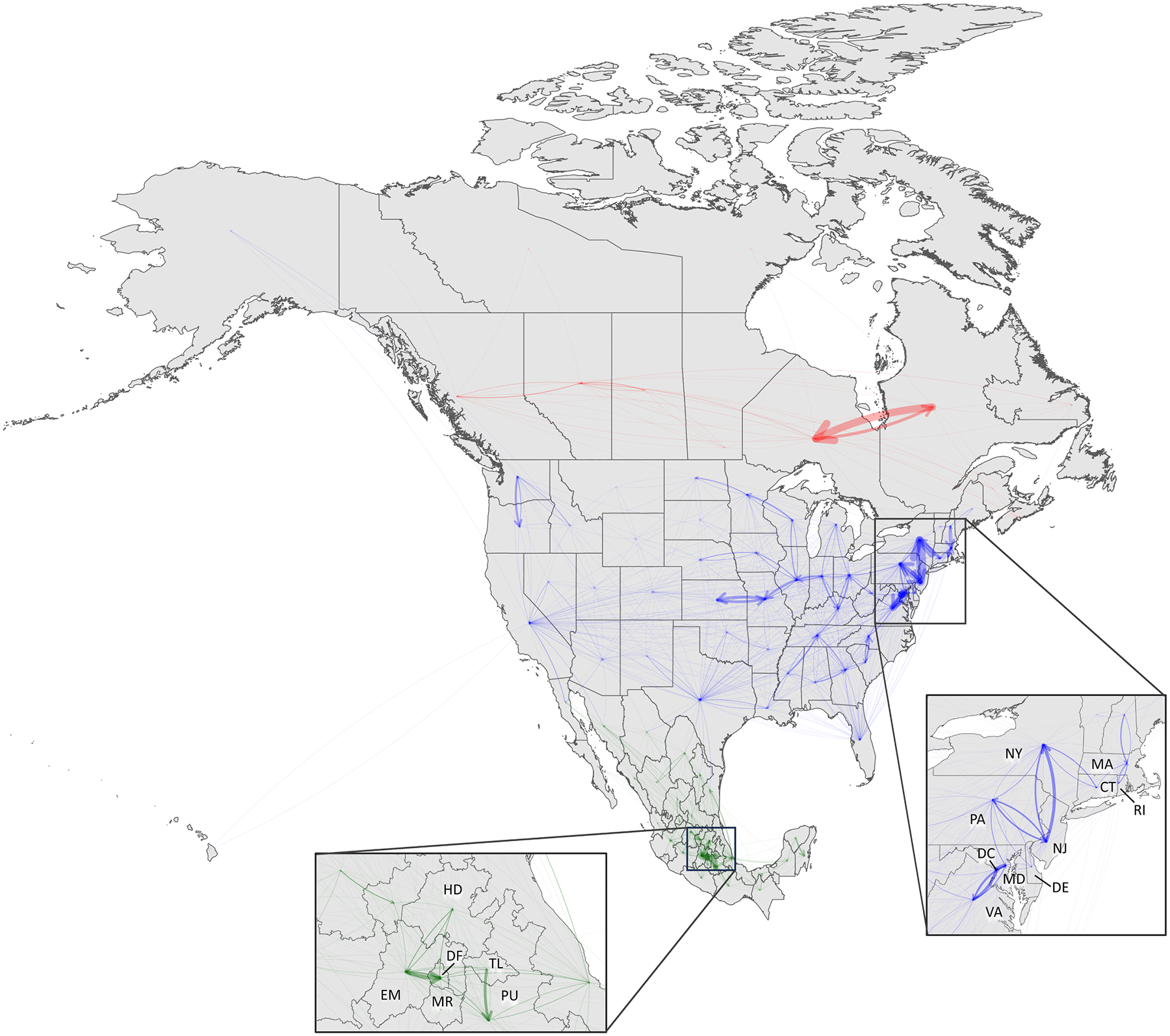
Commuting to work matrix in most of the North American region. The map shows the 10 provinces and 3 territories of Canada, the 50 states and 1 federal district of United States and the 31 states and 1 autonomous city of Mexico that have been used in the model. The arrows represent the flux of individuals commuting daily to work to another location. Arrow size represents the number of commuters, and color represents the country of origin: red for Canada, blue for United States, green for Mexico.

**Fig. 2.**
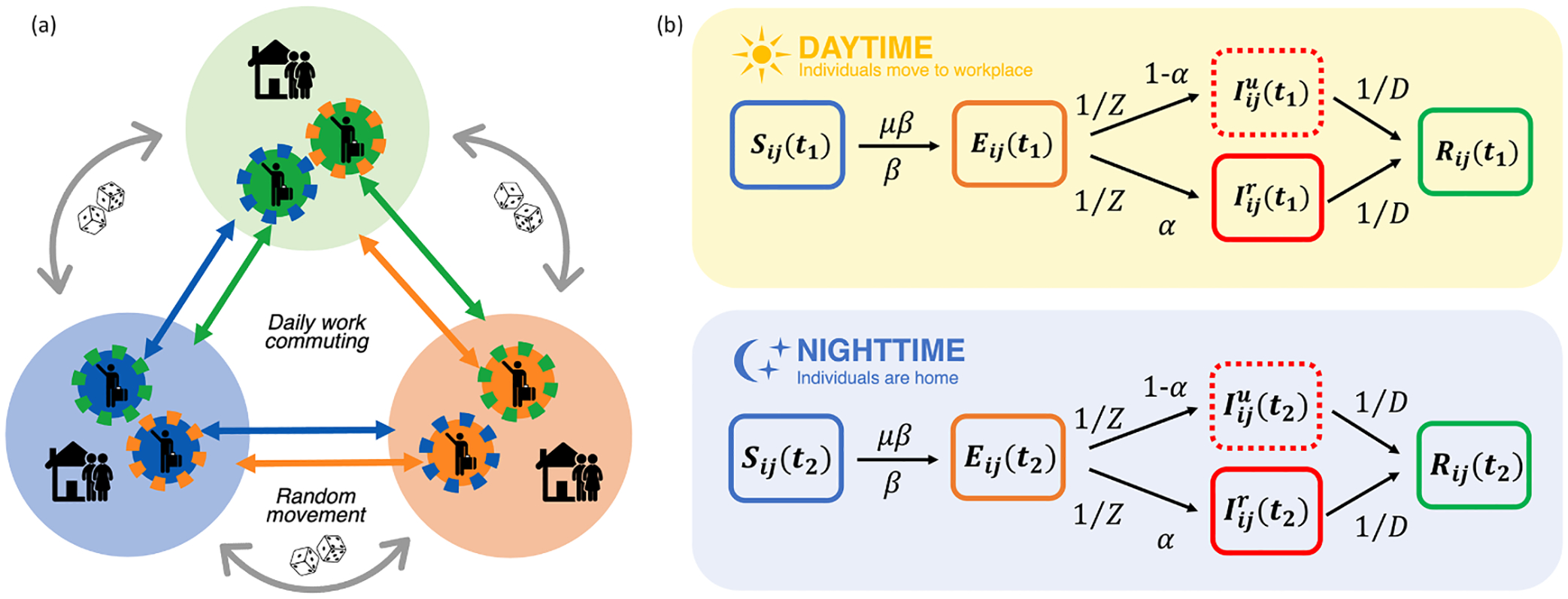
Metapopulation structure and compartmental model: (a) Daily work commuting during the daytime (8 h), some individuals commute from their home to their workplace in another location and mix with the populations present there. During the nighttime (16 h), those commuters return home and mix with other residents who live in the same location. Random movement individuals may travel among locations for reasons other than work. These random visitors circulate among subpopulations following a Markov process, causing a population exchange in all locations **(b)** $I_{ij}^{r}$ reported infected and $I_{ij}^{u}$ unreported infected from location from location j to location i(i←j); $t_{1}$ daytime duration; $t_{2}$ nighttime duration; β transmission rate; α ascertainment rate; μ relative transmissibility of unreported cases; Z latency period; D duration of contagiousness.

We developed a metapopulation susceptible-exposed-infectious-recovered (SEIR) model for the North American region. Specifically, the model represents the 96 first-level administrative divisions of Canada (10 provinces and 3 territories), United States (50 states and 1 federal district) and Mexico (31 states and 1 autonomous city) represented in Fig. 1, with a total population of 483 million (Statistics Canada, 2024; Bureau, 2024; INEGI, 2023). Mixing is simulated as two types of movement: daily commuting and random movement (i.e. all the daily movement across locations due to reasons other than work commuting). Each day, individuals who commute for work interact with two different subpopulations: during the 8-hour workday, they interact with the local subpopulation at their workplace, and during the 16-hour nighttime, they interact with the subpopulation at their place of residence (see Fig. 2b).

### 2.1. Daily commuting matrix

Daily commuting among locations were retrieved and derived from four national datasets: i) Canadian 2016 census (Statistics Canada) Commuting Flow from Geography of Residence to Geography of Work (Government of Canada SC, 2024); ii) Canada Frontier Counts (Statistics Canada): Number of vehicles travelling between Canada and the United States (Statistics Canada, 2023); iii) 2011–2015 5-Year American Community Survey (ACS) Commuting Flows (United States Census Bureau) (US Census Bureau, 2023); and iv) Mexican Intercensal Survey 2015 (National Institute of Statistics and Geography, INEGI) (INEGI, 2023).

The datasets were not directly comparable, and in some instances, commuting flow information was incomplete or missing (e.g., intercountry commuting data). For example, Canada does not provide data on the number of people commuting daily to the U.S. Instead, we used a dataset that counts Canadian and U.S. vehicles crossing the border for same-day trips. Conversely, while cross-country commuter counts between the U.S. and Mexico were available, they lacked information on the specific destination states within the country. The datasets, assumptions, approximations, and methods used to estimate these commuting flows are described in more detail in Supplementary Note 1. The resulting matrix contains the number of people that commute daily to work in another location (states for Mexico and US, provinces or territories for Canada). To account for control measures and closures enacted during the estimation period, the commuting work matrix was scaled based on daily mobility activity, derived from the “change in workplace visitors” trends from Google Community Mobility Reports (COVID-19 Community Mobility Reports Internet, 2023) (see Supplementary Figure 1). To account for movement between locations for purposes other than work commuting, the model includes daily random movement among locations; this movement was set to be proportional to the average number of working commuters among each location pair (see Table 2).

### 2.2. Transmission model

The metapopulation model resolves daytime (8 hours) and nighttime (16 hours) mixing differences, depicting diurnal changes in contact among subpopulations. Transmission occurs as a discrete Markov process during both day and nighttime. To account for the inherent stochasticity in the process, each term in the equation is integrated using a random Poisson process. This approach builds on the framework of previous studies conducted in the U.S (Pei et al., 2020) and is detailed in Eqs. (1)–(10), Table 1, and Supplementary Note 2.

#### Daytime transmission


(1) $$S_{ij}\left(t+dt_{1}\right)=S_{ij}(t)-\text{Pois}\left(\beta_{i}\frac{S_{ij}(t)\sum_{k}I_{ki}^{r}(t)}{N_{i}^{\partial }(t)}dt_{1}\right)-\text{Pois}(\mu \beta_{i}\left.\frac{S_{ij}(t)\sum_{k}I_{ik}^{u}(t)}{N_{i}^{d}(t)}dt_{1}\right)+\text{Pois}\left(\theta dt_{1}\frac{N_{ij}-I_{ij}^{r}(t)}{N_{i}^{d}(t)}\sum_{k\neq i}\frac{{\bar{N}}_{ik}\sum_{l}S_{kl}(t)}{N_{k}^{d}(t)-\sum_{l}I_{lk}^{r}(t)}\right)-\text{Pois}\left(\theta dt_{1}\frac{S_{ij}(t)}{N_{i}^{d}(t)-\sum_{l}I_{li}^{r}(t)}\sum_{k\neq i}{\bar{N}}_{ki}\right)$$

(2) $$E_{ij}\left(t+dt_{1}\right)=E_{ij}(t)+\text{Pois}\left(\beta_{i}\frac{S_{ij}(t)\sum_{k}I_{ki}^{r}(t)}{N_{i}^{d}(t)}dt_{1}\right)+\text{Pois}\left(\mu \beta_{i}\frac{S_{ij}(t)\sum_{k}I_{ki}^{r}(t)}{N_{i}^{d}(t)}dt_{1}\right)-\text{Pois}\left(\frac{E_{ij}(t)}{z}dt_{1}\right)+\text{Pois}\left(\theta dt_{1}\frac{N_{ij}-I_{ij}^{r}(t)}{N_{i}^{d}(t)}\sum_{k\neq i}\frac{{\bar{N}}_{ik}\sum_{l}E_{kl}(t)}{N_{k}^{d}(t)-\sum_{l}I_{lk}^{r}(t)}\right)-\text{Pois}\left(\theta dt_{1}\frac{E_{ij}(t)}{N_{i}^{d}(t)-\sum_{l}I_{li}^{r}(t)}\sum_{k\neq i}{\bar{N}}_{ki}\right)$$

(3) $$I_{ij}^{r}\left(t+dt_{1}\right)=I_{ij}^{r}\left(t\right)+\text{Pois}\left(\alpha \frac{E_{ij}\left(t\right)}{Z}dt_{1}\right)-\text{Pois}\left(\frac{I_{ij}^{r}\left(t\right)}{D}dt_{1}\right)$$

(4) $$I_{ij}^{u}\left(t+dt_{1}\right)=I_{ij}^{u}\left(t\right)+\text{Pois}\left(\left(1-\alpha \right)\frac{E_{ij}\left(t\right)}{Z}dt_{1}\right)-\text{Pois}\left(\frac{I_{ij}^{u}\left(t\right)}{D}dt_{1}\right)+\text{Pois}\left(\theta dt_{1}\frac{N_{ij}-I_{ij}^{r}(t)}{N_{i}^{d}(t)}\sum_{k\neq i}\frac{{\bar{N}}_{ik}\sum_{l}I_{kl}^{u}(t)}{N_{k}^{d}(t)-\sum_{l}I_{lk}^{r}(t)}\right)-\text{Pois}\left(\theta dt_{1}\frac{I_{ij}^{u}(t)}{N_{i}^{d}(t)-\sum_{l}I_{li}^{r}(t)}\sum_{k\neq i}{\bar{N}}_{ki}\right)$$

(5) $$N_{i}^{d}\left(t\right)=N_{ii}+\sum_{k\neq i}I_{ki}^{r}\left(t\right)+\sum_{k\neq i}\left(N_{ik}-I_{ik}^{r}\left(t\right)\right)$$

#### Nighttime transmission


(6) $$S_{ij}(t+1)=S_{ij}\left(t+dt_{1}\right)-\text{Pois}\left(\beta_{j}\frac{S_{ij}\left(t+dt_{1}\right)\sum_{k}I_{kj}^{r}\left(t+dt_{1}\right)}{N_{j}^{n}}dt_{2}\right)-\text{Pois}\left(\mu \beta_{j}\frac{s_{ij}\left(t+dt_{1}\right)\sum_{k}I_{kj}^{u}\left(t+dt_{1}\right)}{N_{j}^{n}}dt_{2}\right)+\text{Pois}\left(\theta dt_{2}\frac{N_{ij}}{N_{j}^{n}}\sum_{k\neq j}\frac{{\bar{N}}_{jk}\sum_{l}S_{lk}\left(t+dt_{1}\right)}{N_{k}^{n}-\sum_{l}I_{lk}^{r}\left(t+dt_{1}\right)}\right)-\text{Pois}\left(\theta dt_{2}\frac{S_{ij}\left(t+dt_{1}\right)}{N_{j}^{n}-\sum_{k}I_{kj}^{r}\left(t+dt_{1}\right)}\sum_{k\neq j}{\bar{N}}_{kj}\right)$$

(7) $$E_{ij}\left(t+1\right)=E_{ij}\left(t+dt_{1}\right)+\text{Pois}\left(\beta_{j}\frac{S_{ij}\left(t+dt_{1}\right)\sum_{k}I_{kj}^{r}\left(t+dt_{1}\right)}{N_{j}^{n}}dt_{2}\right)+\text{Pois}\left(\mu \beta_{j}\frac{S_{ij}\left(t+dt_{1}\right)\sum_{k}I_{kj}^{u}\left(t+dt_{1}\right)}{N_{j}^{n}}dt_{2}\right)-\text{Pois}\left(\frac{E_{ij}\left(t+dt_{1}\right)}{Z}dt_{2}\right)+\text{Pois}\left(\theta dt_{2}\frac{N_{ij}}{N_{j}^{n}}\sum_{k\neq j}\frac{{\bar{N}}_{jk}\sum_{l}E_{lk}\left(t+dt_{1}\right)}{N_{k}^{n}-\sum_{l}I_{lk}^{r}\left(t+dt_{1}\right)}\right)-\text{Pois}\left(\theta dt_{1}\frac{E_{ij}\left(t+dt_{1}\right)}{N_{j}^{n}-\sum_{k}I_{kj}^{r}\left(t+dt_{1}\right)}\sum_{k\neq j}{\bar{N}}_{kj}\right)$$

(8) $$I_{ij}^{r}\left(t+1\right)=I_{ij}^{r}\left(t+dt_{1}\right)+\text{Pois}\left(\alpha \frac{E_{ij}\left(t+dt_{1}\right)}{Z}dt_{2}\right)-\text{Pois}\left(\frac{I_{ij}^{r}\left(t+dt_{1}\right)}{D}dt_{2}\right)$$

(9) $$I_{ij}^{u}(t+1)=I_{ij}^{u}\left(t+dt_{1}\right)+\text{Pois}\left((1-\alpha )\frac{E_{ij}\left(t+dt_{1}\right)}{Z}dt_{2}\right)-\text{Pois}\left(\frac{I_{ij}^{u}\left(t+dt_{1}\right)}{D}dt_{2}\right)+\text{Pois}\left(\theta dt_{2}\frac{N_{ij}}{N_{j}^{n}}\sum_{k\neq j}\frac{{\bar{N}}_{jk}\sum_{l}I_{lk}^{u}\left(t+dt_{1}\right)}{N_{k}^{n}-\sum_{l}I_{lk}^{r}\left(t+dt_{1}\right)}\right)-\text{Pois}\left(\theta dt_{2}\frac{I_{ij}^{u}\left(t+dt_{1}\right)}{N_{j}^{n}-\sum_{k}I_{kj}^{r}\left(t+dt_{1}\right)}\sum_{k\neq j}{\bar{N}}_{ki}\right)$$

(10) $$N_{i}^{n}=\sum_{k}N_{ki}$$

In these equations, $S_{ij}$, $E_{ij}$, $I_{ij}^{r}$, $I_{ij}^{u}$, and $N_{ij}$ represent the susceptible, exposed, reported infectious, unreported infectious, and total population in the subpopulation commuting from location j to location i(i←j) (see Fig. 2b). Vaccines were first introduced in the US during December 2020. By March 31, 2021 (the final day of the simulation), 1.84 % of Canadians, 18.99 % of Americans, and 0.74 % of Mexicans had completed their vaccination protocol (Our World in Data, 2024). Given the limited uptake and the short duration of the simulation, which focused on the initial spread and the first few waves of the pandemic, we assumed that the impact of vaccination, loss of immunity, and reinfection was negligible. In addition, we assumed that no individuals enter or leave the model domain.

The parameters of the model are: β, the transmission rate of reported infections; μ, the relative transmissibility of unreported infections; Z, the average latency period (from infection to contagiousness); D, as the average duration of contagiousness; α, the fraction of documented infections (ascertainment rate); and θ, a multiplicative factor adjusting random movement. A distinct transmission rate, μβ, is defined for undocumented infections: we assume that these individuals show little to no symptoms during infection and are less contagious than documented infections (Li et al., 2020). Each equation is integrated using a Poisson process to capture the stochastic nature of transmission dynamics. In total, the model consists of 3268 metapopulations. To reduce the dimension of the system being estimated and to improve identifiability, we fixed the values of the parameters related to disease progression (Z, D, and μ) and the multiplicative factor for random movement (θ) based on previously works (Pei et al., 2020, 2021). Local transmission rates $\beta_{t}^{l}$ and ascertainment rates $\alpha_{t}^{l}$ are estimated for each of the 96 locations l at time t. The hyperparameter $R_{t}^{l}$, the time-varying reproductive number, was derived using the next-generation matrix approach (Pei et al., 2020; Diekmann et al., 2010) (Eq. (11)).


(11) $$R_{t}^{l}=\beta_{t}^{l}D\left[\alpha_{t}^{l}+\left(1-\alpha_{t}^{l}\right)\mu \right]$$

$R_{t}^{l}$ Time-varying reproductive number for location l at time t

D duration of contagiousness

$\beta_{t}^{l}$ transmission rate of location l at time t

$\alpha_{t}^{l}$ ascertainment rate of location l at time t

μ relative transmissibility of unreported infections

### 2.3. COVID-19 cases and data assimilation

The model is informed by daily COVID-19 confirmed case data retrieved from COVID-19 Open Data — Google Health (COVID-19 Open Data–Google Health Internet, 2023) starting from January 20th 2020 to March 31st 2021 for the 96 locations of Canada, US and Mexico. This time frame was chosen to capture the first three COVID-19 waves (Pei et al., 2021), before the emergence of the Delta and subsequent variants and more widespread uptake of COVID-19 vaccines. A 7-day moving average was applied to the daily cases data to smooth out daily fluctuations in reporting. The smoothing also mitigates the impact of reporting delays or inconsistencies in case data.

The estimation of state variables and parameters is carried out by data assimilation implemented using the Ensemble Adjustment Kalman Filter (EAKF) algorithm (Anderson, 2001), as in previous studies (Shaman et al., 2013; DeFelice et al., 2017). Kalman filters update state variables and parameters using Bayes’ rule, assuming normality for the prior distribution and the likelihood to characterize the posterior distribution only by its mean and covariance. In this model, an ensemble of 300 simulations is integrated to generate a prior distribution of parameters and state variables, including estimation of the observed state variable. The formulas and a more comprehensive explanation of the EAKF are presented in Supplementary Note 3.

### 2.4. Model initialization

Each ensemble member was initialized with a set of parameter and state variable estimates to resemble the epidemiological conditions at the beginning of the SARS-CoV-2 pandemic in 2020. The parameter values related to disease progression (Z, D and μ) were drawn from distributions with ranges reported in Table 2, in accordance with Li et al. (2020). These parameters are assumed to remain fixed over time and to have consistent values across all locations, representing inherent biological characteristics of the disease. The random movement factor θ is also constant over time, with a random value drawn from a uniform distribution between 0 and 0.2 assigned to each location (see Table 2). This value represents the relative volume of random movement compared to commuting, where θ=0.15 indicates that the number of random visitors is 15 % of the average number of commuters between two locations. Exposed (E), reported infectious ($I^{r}$), and unreported infectious ($I^{u}$) individuals were initialized with random draws from a uniform distribution that ranged from 1 to 9 in each subpopulation.

Country-specific initial prior distributions were estimated by leveraging infection-induced seropositivity data (Tang et al., 2022; Jones et al., 2021; Muñoz-Medina et al., 2021) and accounting for significant differences among the three countries in healthcare capacity, testing capacity, and testing strategies (Pei et al., 2020, 2021; Dahal et al., 2021; Hasell et al., 2020; Lee et al., 2021). For the US, the initial prior distribution for the ascertainment rate (α) was specified as a uniform distribution with a mean of 0.25 (see Table 2). This value was derived from estimates of infection-induced seropositivity as of July 2020 (Jones et al., 2021). In contrast, the initial α values for Canada and Mexico, derived from their respective infection-induced seropositivity estimates (Tang et al., 2022; Muñoz-Medina et al., 2021) were found to be near zero with limited range. To address this issue, an exponential distribution shifted to 0.0025 (i.e. the lower bound of α) was assigned as the initial prior distribution for α in Canada and Mexico. (see Table 2). With these adjustments the initial priors reflect the extremely low ascertainment rates observed in these countries at the start of the pandemic while allowing that higher values are possible (more details and calculations in Supplementary Note 4).

The initial values of β were drawn for each location from the normal distribution with mean μ=1.93 and standard deviation σ=0.75 similarly to Li et al. (2020) (see Table 2).

The SEIR-EAKF model-inference system can potentially estimate parameters and state variables values that violate physicality. This could happen, for example, if a state variable or estimated parameter is adjusted by the filter to a value below zero. To avoid this issue, we applied constraints to both the state variables and parameters, subsequently re-assigning values that fell outside specified bounds.

Ensemble members associated with any state variable that exhibited values ≤ 0 were assigned values from the previous day.

The lower bound of α was set to 0.025 corresponding to 5 reported cases per 200 infections. To reflect the differential effort of the countries to increase detection capability, the α lower bound increased over time for Canada and US but remained constant for Mexico (see Supplementary Note 4 for details). The upper bound for α was fixed for all locations at 0.6 (i.e. 60 cases per 100 infections). We allowed the transmission rate β to span a broad range of values, with the lower bound set to 0.2 and the upper bound set to 4. The descriptions, prior ranges and bounds for all the model parameters are shown in Table 2.

### 2.5. System identifiability

To assess the identifiability of the model, we first tested our framework using synthetically generated datasets. Specifically, we generated a suite of synthetic outbreaks using the model (Eqs. (S4)–(S13)) in free simulation, each with arbitrarily assigned values of the epidemiological parameters and initial conditions for state variables. We then ran the full model-inference system 100 times assimilating the daily new reported cases time series generated by each of these free simulations to test the system’s ability to accurately estimate state variable and parameter values. As in practice with actual data, we fixed Z, D, μ and θ, while α and β were estimated for each location. The initial prior distribution and range of the parameters were as reported in Table 2.

## 3. Results

### 3.1. Daily commuting matrix

The daily commuting matrix depicted in Fig. 1 and summarized in Table 3 was obtained by combining and processing the national datasets reported in the Method section and in Supplementary Note 1. Out of a combined population of 483 million, the Canadian population accounts for the 7.3 % (35.1 million), the US for the 67.2 % (324.3 million) and Mexico for the 25.6 % (123.3 million). Around 8.9 million people, or 1.84 % of the represented North American population, commutes daily to another state/province/territory or country to work. Inter-country commuting accounts for the 1.12 % (99,369) of the 8.9 million commuters. Finally, the percentage of internal commuters for each country is similar to the population percentage relative to the total population of the three countries.

### 3.2. System Identifiability

To verify the convergence of the estimated parameters (α and β) to the synthetic truth values created in free simulation, we plotted the model-generated data points over the boxplot distributions of the 3000 estimated parameter values (300 ensemble members × 100 ensemble simulations) at the end of each outbreak among the locations (see Figure S2). Most of the true values fall in the interquartile of the distribution of the estimated values (68 % for α and 84 % for β) while all the true values falls in the 95 % CI of the distribution. This demonstrates the ability of the system to estimate local time-varying α and β values.

### 3.3. Simulation with case data

**Fig. 3.**
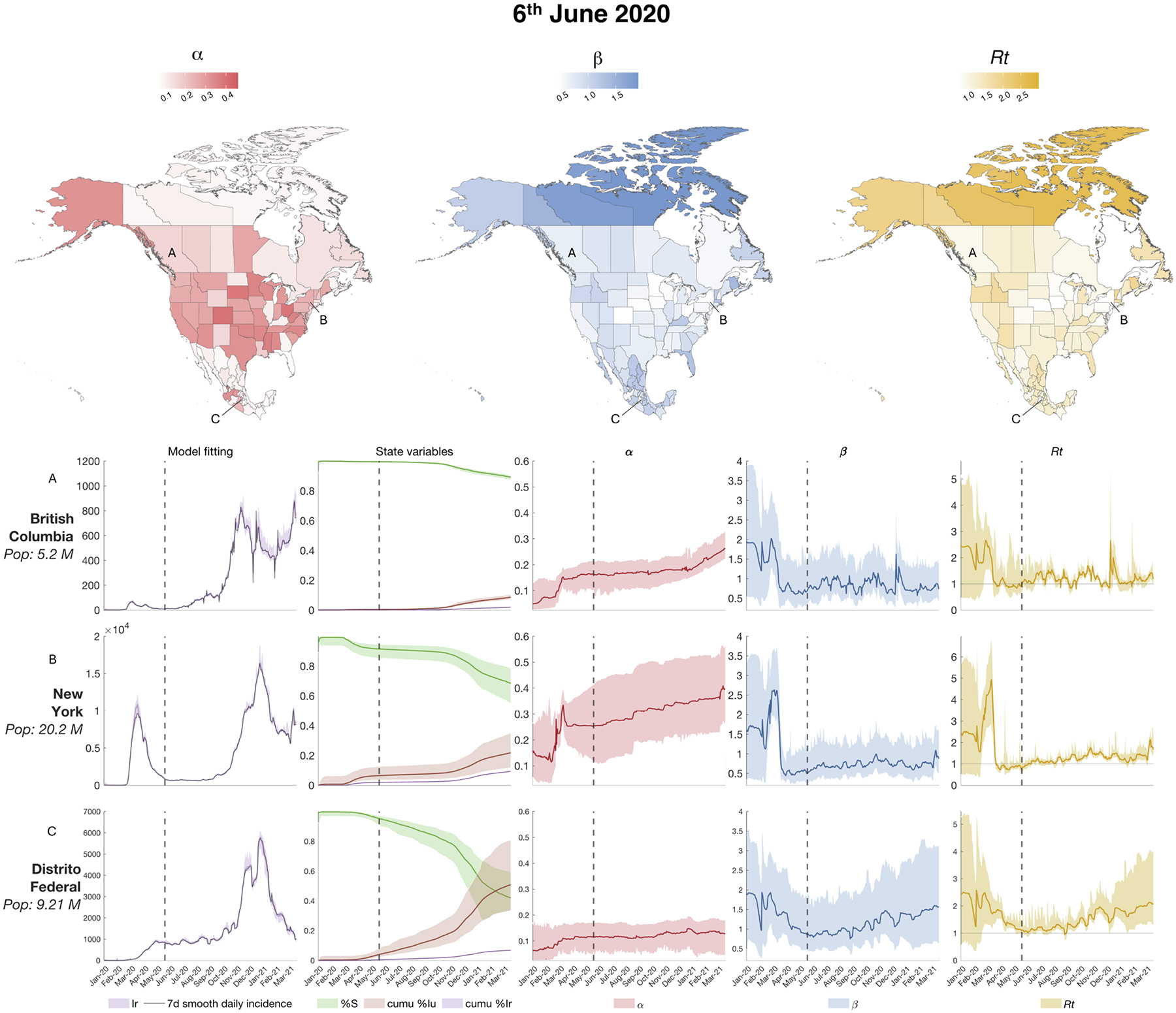
Estimated state variables and parameters on June 6, 2020: the three maps on the top panel show the value of the of the parameters (ascertainment rate α and transmission rate β) and the hyperparameter (basic reproductive number $R_{t}$) for all 96 locations on June 6,2020. The bottom panels show the model fitting (i.e. the estimated observed variable over the 7-day smoothed daily new reported cases), three state variables illustrating disease progression (the susceptible population, cumulative reported infectious and cumulative unreported infectious), and the parameters α, β and $R_{t}$ for 3 selected locations: British Columbia (Canada), New York (US) and Distrito Federal (Mexico City, Mexico). The color shaded areas represent the 95 % credible interval from the 300-member ensemble. The dotted vertical lines indicate the timepoint of reference for the maps.

**Fig. 4.**
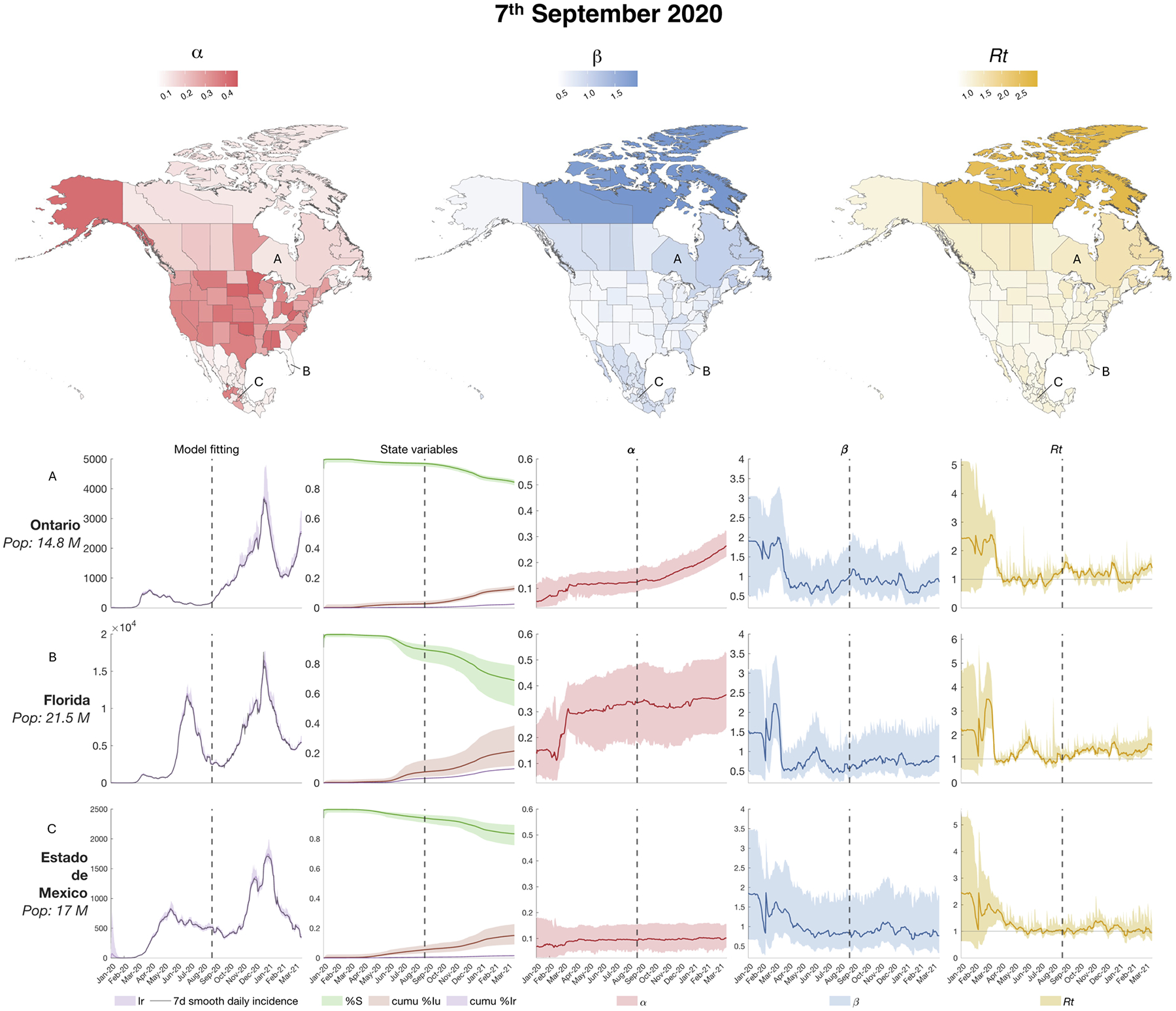
Estimated state variables and parameters on September 7, 2020: the three maps on the top panel show the value of the of the parameters (ascertainment rate α and transmission rate β) and the hyperparameter (basic reproductive number $R_{t}$) for all 96 locations on September 7, 2020. The bottom panels show the model fitting (i.e. the estimated observed variable over the 7-day smoothed daily new reported cases), three state variables illustrating disease progression (the susceptible population, cumulative reported infectious and cumulative unreported infectious), and the parameters α, β and $R_{t}$ for 3 selected locations: Ontario (Canada), Florida (US) and Estado de México (Mexico). The color shaded areas represent the 95 % credible interval from the 300-member ensemble. The dotted vertical lines indicate the timepoint of reference for the maps.

**Fig. 5.**
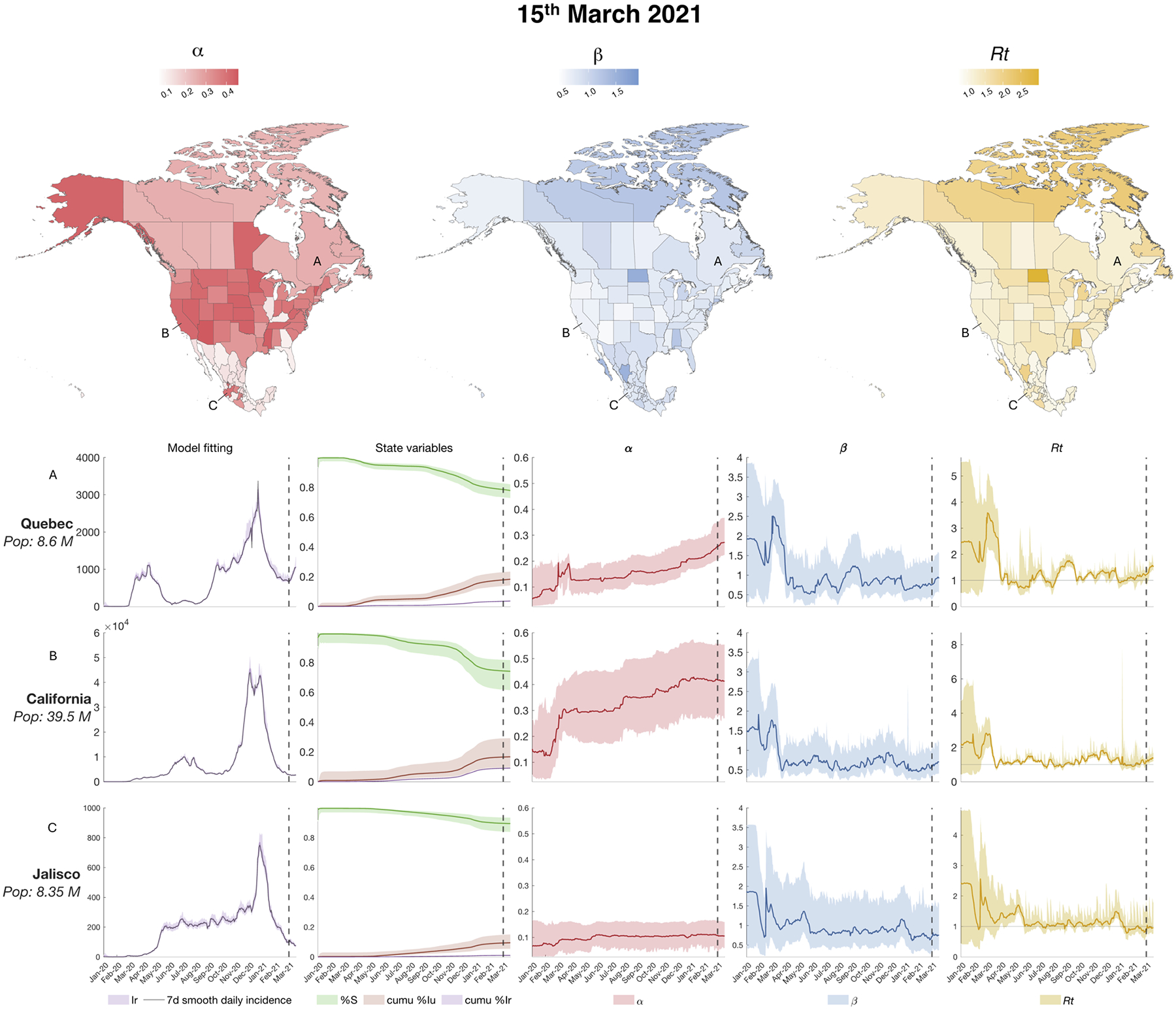
Estimated state variables and parameters on March 15, 2021: the three maps on the top panel show the value of the of the parameters (ascertainment rate α and transmission rate β) and the hyperparameter (basic reproductive number $R_{t}$) for all 96 locations on March 15, 2021. The bottom panels show the model fitting (i. e. the estimated observed variable over the 7-day smoothed daily new reported cases), three state variables illustrating disease progression (the susceptible population, cumulative reported infectious and cumulative unreported infectious), and the parameters α, β and $R_{t}$ for 3 selected locations: Quebec (Canada), California (United States), and Jalisco (Mexico). The color shaded areas represent the 95 % credible interval from the 300-member ensemble. The dotted vertical lines indicate the timepoint of reference for the maps.

The model-inference system was run with the real case data of Canada, United States and Mexico starting from January 20th 2020 to March 31st 2021. The analyses were conducted on a 2023 MacBook Pro with an Apple M3 Pro chip and 18 GB of RAM, with a runtime of 3 minutes and 57 seconds. The three countries experienced asynchronous outbreaks as depicted in Figure S1. To explore disease dynamics over time and location, we selected three timepoints when cases were declining in most of the locations, roughly corresponding to the end of the three pandemic waves experienced in United States during 2020 (Pei et al., 2021). The first wave began in January 2020 and lasted through the spring; we selected June 6, 2020 as the first timepoint. Fig. 3 shows the estimated values of the parameters (α and β) and hyperparameters $\left(R_{t}\right)$ in all 96 locations of the study region at this time point. In addition, the lower panels of Figs. 3–5 show the time progression of model fitting (modeled daily new reported cases and observed daily cases), select state variables (S, cumulative $I^{r}$, cumulative $I^{u}$) and parameter and hyperparameter estimates $\left(\alpha ,\beta ,R_{t}\right)$ for a total of nine selected locations. These locations were selected among the 96 to represent the epidemiological progression in different geographical areas of the North American region, focusing on some of the most populous and epidemiologically relevant locations of the three countries. In Fig. 3 the three selected locations indicated in the maps are British Columbia (Canada), New York (United States) and Distrito Federal (Mexico City, Mexico).

During summer 2020, some locations, particularly in the US, experienced a second wave consisting of a resurgence of cases, with a decline at the beginning of fall. Fig. 4 shows the values of the state variables and parameters on September 7, 2020. The maps show the parameter estimates across all locations, while the temporal dynamics of state variable and parameter estimates are shown for Ontario (Canada), Florida (US) and Estado de México (Mexico).

Most locations experienced a more severe outbreak during the autumn-winter wave of 2020/2021, before the widespread availability of the COVID-19 vaccines. We plot the estimated parameters and hyperparameters on March 15, 2021 in Fig. 5. The state variable and parameter estimate time series are also shown for Quebec (Canada), California (United States), and Jalisco (Mexico).

The sub-national estimated parameter values for each of the three countries were aggregated to the national level using a populationweighted average, and their values on the selected timepoints are reported in Table S3. All estimated mean values of the parameters for all the 96 locations are reported in Table S4.

## 4. Discussion

In this study we developed a SEIR metapopulation model structure and combined it with a data assimilation algorithm (EAKF) to reproduce COVID-19 outbreak dynamics and estimate important epidemiological parameters across most of the North American region during the first three pandemic waves (Pei et al., 2021). The model has demonstrated identifiability in estimating system state variables, as well as the ascertainment rates, α, and transmission rates, β, for the 96 first-level administrative divisions of Canada, United States, and Mexico. The metapopulation structure provided computational efficiency in comparison to agent based models that require HPC (high-performance computing) and cluster computing approaches to run at large scale (Bhattacharya et al., 2024). The transmission module of the model structure relies on the daily work commuting patterns across states, provinces, and territories for disease spreading, with the capability to adjust the daily commuting matrix using Google Mobility Report trends data (COVID-19 Community Mobility Reports Internet, 2023) to account for travel restrictions implemented during the pandemic.

As depicted for selected locations in Figs. 3–5 and reported for all the locations in Table S4, the SEIR-EAKF system estimated large disparities in the ascertainment rates α among the three countries (Pei et al., 2020, 2021; Dahal et al., 2021; Hasell et al., 2020; Lee et al., 2021). Moreover, it estimated a gradual increase in the ascertainment rates α throughout the three major COVID-19 outbreaks during 2020 and at the beginning of 2021 in each country. Specifically, the US showed substantially higher α values in almost all states across the three waves: at the last time point (March 15, 2021) the majority of states had reached an ascertainment rate of ~ 40 %. Canada and Mexico showed smaller ascertainment rates than the US at the end of the estimation: most of the Canadian provinces and territories had an ascertainment rate of ~ 26 %, while Mexican states had less than 16 %. The estimates in the United States are comparable to published estimates of the infection undercount factor derived from a combination of deaths records, confirmed cases, tests, and random surveys published by Irons and Raftery (Irons and Raftery, 2021) (See Figure S4). Note that in Canada and the United States, the increase in α is partially influenced by the increasing lower bound imposed during inference, whereas Mexico has a fixed α lower bound over time.

Transmission rates β remained generally low during the estimation period for the majority of locations. In the United States, the estimated β values in the majority of locations appeared to exhibit minimal variation throughout the estimation period, while in Mexico β generally decreased gradually over time. A few exceptions to the national trends are discussed in the Supplementary Note 5.

The hyperparameter $R_{t}$ (time-varying basic reproductive number) is proportional to β and α, as reported in Eq. (11). The majority of the mean estimated $R_{t}$ values were above the epidemic threshold of $R_{t}=1$ at all the three selected time points and all locations had mean $R_{t}>1$ at least once among the three selected time point. Moreover, the lower estimated mean values for $R_{t}$ never dropped below 0.75. These values suggest a sustained epidemic in the North American region.

Overall, the three countries demonstrated distinct and quite isolated epidemiological histories (See Figs. 3–5 and Table S4). The trends estimated for the parameter and hyperparameter values can be clustered by country, highlighting the national and interconnected epidemiological developments they have undergone. This phenomenon arises from the commuting network structure depicted in Fig. 1, where few arrows traverse national borders, illustrating three major networks (the countries) with limited interconnection.

The model developed for this work uses a new commuting matrix, based on national census data and surveys, to model worker flows in North America. It integrates the SEIR compartmental model with the Ensemble Adjustment Kalman Filter (EAKF), updating state variables and parameters daily based on case data. This approach differs from multi-national models like the GLEAM platform (Balcan et al., 2010; Pastore-Piontti et al., 2016; Zhang et al., 2017), which use commuting and air travel data to create arbitrary airport-centered subpopulations. Instead, in the SEIR-EAKF the subpopulations correspond to the regional boundaries (states for US and Mexico, provinces and territories for Canada), and it uses their work-related commuting data to inform the model. This system focuses more on local transmission dynamics and the role of asymptomatic individuals, whereas GLEAM is able to capture better the global spread of disease that is primarily determined by airline networks. A comprehensive comparison of the approaches is presented in the Supplementary Note 6.

The SEIR-EAKF model has some limitations. For instance, it is not age-stratified, and its commuting matrix is adjusted based solely on work trends, excluding age-specific factors such as school closures, which are known to selectively impact disease dynamics (Viner et al., 2020). On the other hand, the absence of vaccine uptake data in the model is unlikely to have significantly affected the findings, as the model’s time frame ends before COVID-19 vaccines became widely available in the region (Our World in Data, 2024).

The development of multinational dynamical models entails more time and effort compared to localized models because it requires the reconciliation of heterogeneous data sources. For example, the three countries in this study conducted independent census surveys that needed to be carefully interpreted and merged to ensure the resulting multinational contact network was homogeneous. The great advantage of using realistic contact networks that encompass multiple countries is the ability to infer simultaneously across broader regions (e. g. North America), rather than comparing results from separate local inference systems, thus highlighting the geographic spread of the disease across borders. Furthermore, the inference system implemented in this study can serve as a platform for modeling other respiratory infectious diseases, such as influenza, by pairing the North American commuting network developed here with other mathematical models.

Robust models for estimating disease parameters help understand disease dynamics and assess responses across locations. During epidemics, these models can forecast disease spread, aiding health authorities in policymaking. Further, spatially resolved dynamical models can be used compare the effect of public health policies, population density, and mobility patterns across regions. Additionally, this approach supports monitoring systems and counterfactual simulations, enhancing preparedness and enabling data-driven, location-specific interventions to improve epidemic control.

## Supplementary Material

1

## Figures and Tables

**Table 1:** Description of state variables and parameters.

$S_{ij},E_{ij},I_{ij}^{r},I_{ij}^{u},N_{ij}$	Susceptible, exposed, reported infected, unreported infected, and total population in the subpopulations commuting from location j to location i(i←j)
α	ascertainment rate, fraction of documented infections
$\beta_{i}$	Transmission rate of reported infections in state i
μ	Relative transmissibility of unreported infections
Z	Average latency period from Infection to contagiousness
D	Average duration of contagiousness
θ	Multiplicative factor adjusting random movements
${\overset{‾}{N}}_{ij}=\left(N_{ij}+N_{ji}\right)/2$	Average number of commuters between state i and state j
$dt_{1},dt_{2}$	Daytime and nighttime duration $dt_{1}+dt_{2}=1$
$N_{i}^{d},N_{i}^{n}$	Daytime and nighttime population of state i

**Table 2:** parameters description, initial prior distribution, ranges, and parameter type.

	Description	Initial prior range distribution	Bounds	Parameter type
Z	Latency period	U(2,5)days		
D	Duration of contagiousness	U(2,5)days		
μ	Relative transmissibility of unreported infections	U(0.2,0.45)	Fixed	Fixed
θ	Random movement factor	U(0.0,0.2)		
β	Transmission rate	N(1.93,0.75)	[0.2, 4]	
	Ascertainment rate - Canada	Exp(0.022)+0.025		
α	Ascertainment rate – United States	U(0.13,0.37)	[0.025, 0.6]^a^	Estimated
	Ascertainment rate - Mexico	Exp(0.022)+0.025		

a Mexico lower bound is fixed to 0.025 over time; Canada and US lower bound increases by 0.5 % daily: $\alpha_{t+1}^{\text{min}}=\alpha_{t}^{\text{min}}\times 1.005$

**Table 3:** 15 most abundant commuting flows across locations in the North American region.

Residence Location		Work Location		Count
MX	Estado de México	MX	Distrito Federal (Mexico City)	1,059,180
US	New Jersey	US	New York	416,871
US	Maryland	US	District of Columbia (Washington DC)	320,762
CA	Quebec	CA	Ontario	240,660
US	Virginia	US	District of Columbia (Washington DC)	228,039
MX	Tlaxcala	MX	Puebla	175,310
US	New York	US	New Jersey	127,032
US	Maryland	US	Virginia	125,267
US	New Jersey	US	Pennsylvania	125,247
US	Pennsylvania	US	New Jersey	124,808
CA	Ontario	CA	Quebec	104,140
MX	Distrito Federal (Mexico City)	MX	Estado de México	101,260
US	Missouri	US	Kansas	99,778
US	Kansas	US	Missouri	89,681
US	New Hampshire	US	Massachusetts	85,262

## Data Availability

The model scripts and the input data are publicly available on GitHub https://github.com/MatteoPS/NA_SEIR-EAKF
