# Supplementary material for: Modelling COVID-19 in the North American region with a metapopulation network and Kalman filter
Source: Epidemics. Author manuscript; Available in PMC 2025 Apr 24. (PMC12020409; doi:10.1016/j.epidem.2025.100818)
Supplement: 1 [file NIHMS2066145-supplement-1.docx]

**Modelling COVID-19 in the North American region with a metapopulation network and Kalman filter**

Matteo Perini^a*^, Teresa K. Yamana^a^, Marta Galanti^a^, Jiyeon Suh^a^, Roselyn Kaondera-Shava^a^, Jeffrey Shaman^a,b^

*^a^Department of Environmental Health Sciences, Mailman School of Public Health, Columbia University, 722 W 168th St, New York, NY, 10032 United States of America
^b^Columbia Climate School, Columbia University, Level A Hogan, 2910 Broadway, New York, NY, 10025 United States of America*

* Corresponding author:
Matteo Perini [matteo.perini@columbia.edu](mailto:matteo.perini@columbia.edu)

[Supplementary Note 1: 1](#_Toc184067056)
Detailed description of the commuting matrix

[Supplementary Table 1 5](#_Toc184067057)

[Supplementary Note 2 5](#_Toc184067058)
Metapopulation SEIR dynamical model

[Supplementary Table 2 7](#_Toc184067059)

[Supplementary Note 3: 9](#_Toc184067060)
Ensemble Adjustment Kalman Filter (EAKF)

[Supplementary Note 4 11](#_Toc184067061)
Ascertainment rate initialization and lower bound

[Supplementary Note 5 13](#_Toc184067062)
Exceptions to the national trends in the estimated parameters

[Supplementary Note 6 14](#_Toc184067063)
SEIR-EAKF comparison with GLEAM platform

[Supplementary Figure 1 16](#_Toc184067064)

[Supplementary Figure 2 17](#_Toc184067065)

[Supplementary Figure 3 18](#_Toc184067066)

[Supplementary Figure 4 19](#_Toc184067067)

[Supplementary Table 3 20](#_Toc184067068)

[Supplementary Table 4 21](#_Toc184067069)

[References 24](#_Toc184067070)

Supplementary Note 1:

**Detailed description of the commuting matrix**

*Description of the datasets*

The commuting matrix of the North American region was generated using 4 national datasets:

1. *Canadian 2016 census (Statistics Canada): Commuting Flow from Geography of Residence to Geography of Work* [1]:

This dataset provides the average number of commuters among Canadian Census Divisions (CD). These raw data were aggregated to obtain the commuting flow at province and territory level. This dataset lacks information regarding the inter-country commuting (i.e. Canadian citizens crossing the American border to go to work).

1. *Canada Frontier Counts (Statistics Canada): Number of vehicles travelling between Canada and the United States* [2]*:*

This dataset provides a count of monthly vehicles entering Canada via land customs from 1970 to 2021. The data are divided by province/territory, mode of transportation (e.g. automobiles, trucks, other), length of stay (e.g. same day or single night) and vehicle origin (United States vehicles entering or Canadian vehicles returning). For this work, we filtered the 2015 data for United States automobiles entering and Canadian automobiles returning to each province/territory with length of stay equal to one day.

1. *2011-2015 5-Year American Community Survey (ACS) Commuting Flows (United States Census Bureau)* [3]:

This dataset provides the commuting flows for the United States and Puerto Rico averaged over the years 2011-2015. The dataset is provided at state and county level. The data specify for each county or state how many people commute to another county, state, or country. Estimates are obtained by pooling together the results of five years (2011-2015).

1. *Mexican Intercensal Survey 2015 (National Institute of Statistics and Geography, INEGI)* [4]:
   This dataset contains the raw results of the Mexican Intercensal Survey 2015. Presently, it is the only national survey to provide information on commuting patterns of Mexican residents. Data are derived from a sampling of roughly 10% of the population (2.9 million commuters, representing 32 million workers in Mexico as stated in a report from the “Cooperative Mobility for Competitive Megaregions” at The University of Texas at Austin [5] ). The average number of daily commuters for each Mexican state was obtained by aggregating the answers from the question: “*In which State (or Country) is the business, company or place where you worked last week?*”. The assumption is that anyone replying to this question would commute daily in that State/Country. The resulting counts were multiplied by 10 to scale up to include the unsampled population.

*Commuting flow among the Countries:*

We only considered the movements between bordering or "near-bordering" locations across countries (i.e. states/provinces/territories less than ~150 miles from a national border). Note, the datasets report a considerable number of people having their place of work in locations of another country that do not share a border or that is not close to their residence state; however, such distances generally make daily commuting unfeasible and they have been disregarded form the calculations of the commuting flows.

For USA and Mexico, the datasets provide the number of commuters from each state to other countries, but they do not report the destination state, i.e. they show the total number of people entering another country to work but do not specify which state within the foreign country. To distribute the commuters among the states of Mexico and USA (and vice versa) we used the formula:

1. $C_{l^{A}\to m^{B}}=C_{l^{A}\to B}\frac{C_{m^{B}\to A}}{C_{B\to A}}$

$C_{l^{A}\to m^{B}}$ commuters from state $l$ of country $A$ to state $m$ of country $B$
$C_{l^{A}\to B}$ commuters from state $l$ of country $A$ to all the neighboring states of country $B$

$C_{m^{B}\to A}$ commuters from state $m$ of country $B$ to all the neighboring states of country $A$

$C_{B\to A}$ commuters from all the neighboring states of country $B$ to country $A$

Using this formula, we represent the non-homogeneous nature of the passage across the US-Mexican border. All commuting occurs through the relatively few custom stations that are non-uniformly distributed among the border states of Mexico and the US.

The Canadian census does not provide information on the numbers of individuals commuting to other countries, whereas the US provides the number of commuters from each US state to Canada without specifying the province or territory of destination. To approximate the number of daily commuters from Canada to US, we combined:

- The Canadian frontier counts of vehicles crossing the border (at province/territory level to or from US)
- The US dataset of commuters to Canada (at state level to Canada).

Because people cross borders by car for reasons other than commuting, the number of vehicles crossing the border differs from the number of commuters between the two countries. We assumed that the ratio of cars to commuters crossing the border is maintained in both directions:

1. $\frac{C_{CA\to US}}{V_{CA\to US}}=\frac{C_{US\to CA}}{V_{US\to CA}}$

$C_{CA\to US}$ Total daily commuters from Canada to US

$V_{CA\to US}=5,546$ Daily Canadian vehicles (cars) re-entering Canada after a one-day trip

(daily average from Canadian frontier counts of 2015)

$C_{US\to CA}= 4,605$ Total daily commuters form US to Canada
(from 2011-2015 US census)

$V_{US\to CA}= 13,017$ Daily US vehicles (cars) entering Canada for a one-day trip

(daily average from Canadian frontier counts of 2015)

Solving (S2) for $C_{CA\to US}$ we obtain:

1. $C_{CA\to US}=V_{CA\to US}\frac{C_{US\to CA}}{V_{US\to CA}}\approx1,962$

The US commuters to Canada accounts for around 35% of the total US vehicles crossing the Canadian border daily. Therefore, we multiplied the number of Canadian cars crossing the US border daily by the same factor.

Finally, the total US commuters to Canada were distributed to the Canadian provinces/territories using formula (S1), where $C_{m^{B}\to A}$are the Canadian commuters to the US from each province or territory and $C_{B\to A}$ is the total Canadian commuters to US.

Supplementary Table 1*: Number of commuters by country of residence and workplace. Individuals with residence and workplace in the same country are commuting between states/provinces/territories within that country. The last column shows the percentage over the total commuters. The last row shows the total number of cross-border (state, province, territory, or country) commuters and its percentage over the total population of the model.*

| **Residence** | **Workplace** | **Commuters** | **% over Total**  **Commuters** |
| --- | --- | --- | --- |
| Canada | Canada | 527,300 | 5.93 |
| Canada | USA | 1,962 | 0.02 |
| USA | Canada | 4,605 | 0.05 |
| USA | USA | 5,334,072 | 60.02 |
| USA | Mexico | 12,892 | 0.15 |
| Mexico | USA | 79,910 | 0.90 |
| Mexico | Mexico | 2,925,820 | 32.92 |
|  |  | **Total**  **Commuters** | **% over total**  **Population** |
|  |  | 8,890,144 | 1.84 |

Supplementary Note 2**:**

**Metapopulation SEIR dynamical model**

In this work we developed a metapopulation SEIR model to simulate COVID-19 transmission across the North American Region, encompassing Canadian provinces and territories, US states, and Mexican states. The model accounts for both documented and undocumented infections, with separate transmission rates defined for each. We assume no reinfection with SARS-CoV-2 and no vaccine effect on population susceptibility given their deployment on general population in the first months of 2021. Our model incorporates two forms of human mobility across the 96 locations: regular daily commuting and diffusive random movement. Commuters travel to their workplaces during the day, interacting with the local population, and return home in the evening, mixing with individuals in their residential areas. Besides commuting, a portion of the population in each location, proportional to inter-location commuters, travels for non-work purposes in random locations. As population dynamics differ between daytime and nighttime, we separately model COVID-19 transmission dynamics for these periods. This involves formulating transmission as a discrete Markov process and employing random sampling from Poisson distributions to capture stochastic model dynamics. The transmission dynamics are expressed through the following equations, the description of variables and parameters can be found in **Supplementary Table 2**.

**Daytime transmission**

1. $S_{ij}\left( t + {dt}_{1} \right)=S_{ij}\left( t \right)-Pois\left( \beta_{i}\frac{S_{ij}\left( t \right)\sum_{k} I_{ki}^{r}\left( t \right)}{N_{i}^{d}\left( t \right)}{dt}_{1} \right)-Pois\left( \mu\beta_{i}\frac{S_{ij}\left( t \right)\sum_{k} I_{ik}^{u}\left( t \right)}{N_{i}^{d}\left( t \right)}{dt}_{1} \right)+Pois\left( \theta{dt}_{1}\frac{N_{ij}-I_{ij}^{r}\left( t \right)}{N_{i}^{d}\left( t \right)}\sum_{k\neq i} \frac{\bar{N}_{ik}\sum_{l} S_{kl}\left( t \right)}{N_{k}^{d}\left( t \right)-\sum_{l} I_{lk}^{r}\left( t \right)} \right)-Pois\left( {\theta dt}_{1}\frac{S_{ij}\left( t \right)}{N_{i}^{d}\left( t \right)-\sum_{l} I_{li}^{r}\left( t \right)}\sum_{k\neq i} \bar{N}_{ki} \right)$
2. $E_{ij}\left( t + {dt}_{1} \right)=E_{ij}\left( t \right)+ Pois\left( \beta_{i}\frac{S_{ij}\left( t \right)\sum_{k} I_{ki}^{r}\left( t \right)}{N_{i}^{d}\left( t \right)}{dt}_{1} \right)+Pois\left( {\mu\beta}_{i}\frac{S_{ij}\left( t \right)\sum_{k} I_{ik}^{u}\left( t \right)}{N_{i}^{d}\left( t \right)}{dt}_{1} \right)-Pois\left( \frac{E_{ij}\left( t \right)}{Z}{dt}_{1} \right)+Pois\left( \theta{dt}_{1}\frac{N_{ij}-I_{ij}^{r}\left( t \right)}{N_{i}^{d}\left( t \right)}\sum_{k\neq i} \frac{\bar{N}_{ik}\sum_{l} E_{kl}\left( t \right)}{N_{k}^{d}\left( t \right)-\sum_{l} I_{lk}^{r}\left( t \right)} \right)-Pois\left( {\theta dt}_{1}\frac{E_{ij}\left( t \right)}{N_{i}^{d}\left( t \right)-\sum_{l} I_{li}^{r}(t)}\sum_{k\neq i} \bar{N}_{ki} \right)$
3. $I_{ij}^{r}\left( t + {dt}_{1} \right)= I_{ij}^{r}\left( t \right)+Pois\left( \alpha\frac{E_{ij}\left( t \right)}{Z}{dt}_{1} \right)-Pois\left( \frac{I_{ij}^{r}\left( t \right)}{D}{dt}_{1} \right)$
4. $I_{ij}^{u}\left( t + {dt}_{1} \right)= I_{ij}^{u}\left( t \right)+Pois\left( \left( 1- \alpha\right)\frac{E_{ij}\left( t \right)}{Z}{dt}_{1} \right)-Pois\left( \frac{I_{ij}^{u}\left( t \right)}{D}{dt}_{1} \right)+Pois\left( \theta{dt}_{1}\frac{N_{ij}-I_{ij}^{r}\left( t \right)}{N_{i}^{d}(t)}\sum_{k\neq i} \frac{\bar{N}_{ik}\sum_{l} I_{kl}^{u}(t)}{N_{k}^{d}\left( t \right)-\sum_{l} I_{lk}^{r}(t)} \right)-Pois\left( {\theta dt}_{1}\frac{I_{ij}^{u}\left( t \right)}{N_{i}^{d}\left( t \right)-\sum_{l} I_{li}^{r}(t)}\sum_{k\neq i} \bar{N}_{ki} \right)$
5. $N_{i}^{d}\left( t \right)=N_{ii}+\sum_{k\neq i} I_{ki}^{r}\left( t \right)+\sum_{k\neq i} {(N_{ik}-I}_{ik}^{r}\left( t \right))$

**Nighttime transmission**

1. $S_{ij}\left( t + 1 \right)=S_{ij}\left( t+{dt}_{1} \right)-Pois\left( \beta_{j}\frac{S_{ij}\left( t+{dt}_{1} \right)\sum_{k} I_{kj}^{r}\left( t+{dt}_{1} \right)}{N_{j}^{n}}{dt}_{2} \right)-Pois\left( \mu\beta_{j}\frac{S_{ij}\left( t+{dt}_{1} \right)\sum_{k} I_{kj}^{u}\left( t+{dt}_{1} \right)}{N_{j}^{n}}{dt}_{2} \right)+Pois\left( \theta{dt}_{2}\frac{N_{ij}}{N_{j}^{n}}\sum_{k\neq j} \frac{\bar{N}_{jk}\sum_{l} S_{lk}\left( t+{dt}_{1} \right)}{N_{k}^{n}-\sum_{l} I_{lk}^{r}\left( t+{dt}_{1} \right)} \right)-Pois\left( {\theta dt}_{2}\frac{S_{ij}\left( t+{dt}_{1} \right)}{N_{j}^{n}-\sum_{k} I_{kj}^{r}(t+{dt}_{1})}\sum_{k\neq j} \bar{N}_{kj} \right)$
2. $E_{ij}\left( t + 1 \right)=E_{ij}\left( t+{dt}_{1} \right)+ Pois\left( \beta_{j}\frac{S_{ij}\left( t+{dt}_{1} \right)\sum_{k} I_{kj}^{r}\left( t+{dt}_{1} \right)}{N_{j}^{n}}{dt}_{2} \right)+Pois\left( {\mu\beta}_{j}\frac{S_{ij}\left( t+{dt}_{1} \right)\sum_{k} I_{kj}^{u}\left( t+{dt}_{1} \right)}{N_{j}^{n}}{dt}_{2} \right)-Pois\left( \frac{E_{ij}\left( t+{dt}_{1} \right)}{Z}{dt}_{2} \right)+Pois\left( \theta{dt}_{2}\frac{N_{ij}}{N_{j}^{n}}\sum_{k\neq j} \frac{\bar{N}_{jk}\sum_{l} E_{lk}\left( t+{dt}_{1} \right)}{N_{k}^{n}-\sum_{l} I_{lk}^{r}\left( t+{dt}_{1} \right)} \right)-Pois\left( {\theta dt}_{1}\frac{E_{ij}\left( t+{dt}_{1} \right)}{N_{j}^{n}-\sum_{k} I_{kj}^{r}(t+{dt}_{1})}\sum_{k\neq j} \bar{N}_{kj} \right)$
3. $I_{ij}^{r}\left( t + 1 \right)= I_{ij}^{r}\left( t+{dt}_{1} \right)+Pois\left( \alpha\frac{E_{ij}\left( t+{dt}_{1} \right)}{Z}{dt}_{2} \right)-Pois\left( \frac{I_{ij}^{r}\left( t+{dt}_{1} \right)}{D}{dt}_{2} \right)$
4. $I_{ij}^{u}\left( t + 1 \right)= I_{ij}^{u}\left( t+{dt}_{1} \right)+Pois\left( \left( 1- \alpha\right)\frac{E_{ij}\left( t+{dt}_{1} \right)}{Z}{dt}_{2} \right)-Pois\left( \frac{I_{ij}^{u}\left( t+{dt}_{1} \right)}{D}{dt}_{2} \right)+Pois\left( \theta{dt}_{2}\frac{N_{ij}}{N_{j}^{n}}\sum_{k\neq j} \frac{\bar{N}_{jk}\sum_{l} I_{lk}^{u}\left( t+{dt}_{1} \right)}{N_{k}^{n}-\sum_{l} I_{lk}^{r}\left( t+{dt}_{1} \right)} \right)-Pois\left( {\theta dt}_{2}\frac{I_{ij}^{u}\left( t+{dt}_{1} \right)}{N_{j}^{n}-\sum_{k} I_{kj}^{r}(t+{dt}_{1})}\sum_{k\neq j} \bar{N}_{ki} \right)$
5. $N_{i}^{n}=\sum_{k} N_{ki}$

Supplementary Table 2*: Description of state variables and parameters*

| $S_{ij}$, $E_{ij}$*,* $I_{ij}^{r}$*,* $I_{ij}^{u}$*,* $N_{ij}$ | Susceptible, exposed, reported infected, unreported infected, and total population in the subpopulations commuting from location *j* to location *i* (*i* ←*j*) |
| --- | --- |
| $\alpha$ | ascertainment rate, fraction of documented infections |
| $\beta_{i}$ | Transmission rate of reported infections in state *i* |
| $\mu$ | Relative transmissibility of unreported infections |
| $Z$ | Average latency period from Infection to contagiousness |
| $D$ | Average duration of contagiousness |
| $\theta$ | Multiplicative factor adjusting random movements |
| $\bar{N}_{ij}=(N_{ij}$ + $N_{ji})/2$ | Average number of commuters between state *i* and state *j* |
| ${dt}_{1}$, ${dt}_{2}$ | Daytime and nighttime duration $dt_{1}+dt_{2}=1$ |
| $N_{i}^{d}$, $N_{i}^{n}$ | Daytime and nighttime population of state *i* |

To assess changes in disease transmission over time due to factors like public health interventions, behavior changes, and population immunity we calculated the time-varying reproductive number $R_{t}^{l}$ for each location. It represents the average number of secondary infections caused by an infected individual at a specific time during an epidemic. We derived $R_{t}^{l}$using the next-generation matrix approach as described by Diekmann et al. [6]

1. $R_{t}^{l}=\beta_{t}^{l}D[\alpha_{t}^{l}+\left( 1-\alpha_{t}^{l} \right)\mu]$

$R_{t}^{l}$ Time-varying reproductive number for location $l$ at time $t$

$D$ Duration of contagiousness

$\beta_{t}^{l}$ Transmission rate of location $l$ at time $t$

$\alpha_{t}^{l}$ ascertainment rate of location $l$ at time $t$

$\mu$ relative transmissibility of unreported infections

We assume that the portion of the infectious population that is reported, $I_{ij}^{r}$, remains immobile and does not partake in human movement. Additionally, we assume that no individuals enter or exit the model, and immunity acquired after initial infection remains unchanged throughout the relatively brief simulation period. For this reason, we can compute the $R_{ij}$ (the removed population) as $R_{ij}=\sum_{1}^{t} I_{ij}^{r}+\sum_{1}^{t} I_{ij}^{u}$ which is the cumulative sum of daily new reported and unreported cases, or the sum of the individuals that exited the two infectious compartments (**Figure 2**). We integrate **Eqs.** **S4-S13** using a Poisson process to represent the stochastic nature of the transmission process. During the daytime, $N_{ij}$ individuals residing in location 𝑗 commute to workplace 𝑖, interacting with the local population. At nighttime, these commuters return home and mix with other residents in location 𝑗. Additionally, individuals engage in random movement between locations for purposes other than work, circulating among subpopulations following a Markov process and contributing to population exchange across all locations.

**Daily work commuting**. For the daily work commuting, the daytime population in location 𝑖, $N_{i}^{d}\left( t \right)=N_{ii}+\sum_{k\neq i} I_{ki}^{r}\left( t \right)+\sum_{k\neq i} {(N_{ik}-I}_{ik}^{r}\left( t \right))$, is composed of individuals who both live and work in 𝑖, reported infected individuals who would otherwise commute to other locations $k (k \neq i)$, and individuals commuting to 𝑖 from other location $k (k \neq i)$ who are not reported infections. New infections of any subpopulation $N_{ij}$ occurs through contact with documented and with undocumented infectious individuals. Specifically, for each susceptible individual at time t $S_{ij}(t)$, the probability of encountering a documented infectious person is $\sum_{k} I_{ki}^{r}\left( t \right)/N_{i}^{d}\left( t \right)$, where $\sum_{k} I_{ki}^{r}\left( t \right)$ is the number of documented infectious individuals in location $i$. These encounters create $\beta_{i}\frac{S_{ij}\left( t \right)\sum_{k} I_{ki}^{r}\left( t \right)}{N_{i}^{d}\left( t \right)}{dt}_{1}+\mu\beta_{i}\frac{S_{ij}\left( t \right)\sum_{k} I_{ik}^{u}\left( t \right)}{N_{i}^{d}\left( t \right)}{dt}_{1}$new infections during the daytime period ${dt}_{1}$, as indicated in **Eq. S4-S5**. This term accounts for the mixing of populations from different locations due to work commuting and represents intra-location transmission during the daytime in location 𝑖.

**Random movement.** In addition to people commuting for work, during daytime, $\theta{dt}_{1}\bar{N}_{ik}$ people are drawn uniformly from population $k (k\neq i)$ and are randomly distributed to the subpopulation of location $i$ (reported documented infectious individuals are not drawn as they stay home). This exchange exists for each pair of locations. First, we compute the number of susceptible individuals entering the subpopulation $S_{ij}(t)$. In the other locations $k (k\neq i)$ the probability that a random visitor is susceptible is $\sum_{l} S_{kl}(t)/(N_{k}^{d}\left( t \right)-\sum_{l} I_{lk}^{r}\left( t \right))$ where $\sum_{l} S_{kl}(t)$ is the susceptible individuals that moved to location $k$ form any location $l$, while $N_{k}^{d}\left( t \right)-\sum_{l} I_{lk}^{r}\left( t \right)$ is the total population minus the reported infected in location $k$. Therefore, the total susceptible population entering location $i$ is $\theta{dt}_{1}\bar{N}_{ik}\sum_{k\neq i} \sum_{l} S_{kl}(t)/(N_{k}^{d}\left( t \right)-\sum_{l} I_{lk}^{r}\left( t \right))$. These individuals are redistributed into the subpopulation of location $i$, here the people in the subpopulation $N_{ij}$ is $(N_{ij}-I_{ij}^{r}\left( t \right))/N_{i}^{d}\left( t \right)$. Finally, the susceptible individuals entering $S_{ij}(t)$ for the random movement can be computed as: $\theta{dt}_{1}\frac{N_{ij}-I_{ij}^{r}\left( t \right)}{N_{i}^{d}\left( t \right)}\sum_{k\neq i} \frac{\bar{N}_{ik}\sum_{l} S_{kl}\left( t \right)}{N_{k}^{d}\left( t \right)-\sum_{l} I_{lk}^{r}\left( t \right)}$.

To compute the number of individuals leaving $S_{ij}(t)$ through the random movement process, we computed the number of individuals leaving location $i$: $\theta{dt}_{1}\sum_{k\neq i} \bar{N}_{ki}$. Then, the computed the susceptible people coming from $N_{ij}$ is $S_{ij}(t)/(N_{i}^{d}\left( t \right)-\sum_{l} I_{li}^{r}\left( t \right))$. The resulting number of susceptible individuals leaving $S_{ij}(t)$ is thus ${\theta dt}_{1}\frac{S_{ij}\left( t \right)}{N_{i}^{d}\left( t \right)-\sum_{l} I_{li}^{r}\left( t \right)}\sum_{k\neq i} \bar{N}_{ki}$.

The random movement (i.e. exchange of individuals) among other compartments can be computed similarly. **Eq. S6** does not have terms related to random movement: reported infectious individuals are assumed not to move from their subpopulation. Similar to daytime, **Eq. S9-S13** represent transmission during nighttime.

# Supplementary Note 3:

**Ensemble Adjustment Kalman Filter (EAKF)**

The ensemble adjustment Kalman filter (EAKF) was originally designed for weather forecasting and operates under the assumption that both the prior and the likelihood follow a normal Gaussian distribution. It deterministically transforms the prior distribution into a posterior using Bayes’ rule. The EAKF represents the state-space distribution using an ensemble of system state vectors as samples. The assumption of Gaussian distributions for both the prior and likelihood enables characterization by their first two moments, the mean and variance. At each observation time point, the model is halted, and the ensemble and observations are used to calculate the Kalman gain, which is used to update the observed state variable. The unobserved state variables and parameters are then updated in proportion to the same Kalman gain. Finally, the posterior estimates are used as priors and model integration through time continues to the next observation.

The ensemble members update occurs following Bayes’ rule (posterior ∝ prior x likelihood). More specifically, the process involves the convolution of the two Gaussian distributions. The observed state variable (daily reported cases) are updated deterministically so that the higher moments of the prior distribution are preserved in the posterior. The *i*th ensemble member is updated at each timestep as:

1. $o_{t, post}^{i}=\frac{\sigma_{t, obs}^{2}}{\sigma_{t, obs}^{2}+\sigma_{t,prior}^{2}}\bar{o}_{t,prior}+\frac{\sigma_{t, prior}^{2}}{\sigma_{t, obs}^{2}+\sigma_{t,prior}^{2}}y_{t}+\sqrt{\frac{\sigma_{t, obs}^{2}}{\sigma_{t, obs}^{2}+\sigma_{t,prior}^{2}}}(o_{t, prior}^{i}-\bar{o}_{t,prior})$

$o_{t, post}^{i}$ Posterior of the observed variable

$o_{t, prior}^{i}$ Prior of the observed variable for the *i*th ensemble member at time *t*

$\bar{o}_{t,prior}$ Mean of the prior observed variable at time *t*

$\sigma_{t, obs}^{2}$ Variance of the observed variable

$\sigma_{t, prior}^{2}$ Variance of the prior observed variable

$y_{t}$ Observation at time at time *t*

The unobserved variables and parameters are also updated daily using their covariability with the observed variable. Their covariability can be computed directly from the ensemble. The *i*th ensemble member of an unobserved state variable or unobserved parameter is update at each timestep as:

1. $x_{t,post}^{i}=x_{t,prior}^{i}+\frac{\sigma\left( \left\{ x_{t,prior} \right\}_{n},\left\{ o_{t,prior} \right\}_{n} \right)}{\sigma_{t, prior}^{2}}(o_{t,post}^{i}-o_{t, prior}^{i})$

$x_{t,post}^{i}$ Posterior of unobserved state variable or parameter for the *i*th ensemble member at time *t*

$x_{t,prior}^{i}$ Prior of unobserved state variable or parameter for the *i*th ensemble member at time *t*

$\sigma\left( \left\{ x_{t,prior} \right\}_{n},\left\{ o_{t,prior} \right\}_{n} \right)$ Covariance between the priors of the of unobserved state variable or parameter and the observed state variable

The Susceptible population $S_{ij}$ is not directly adjusted by the EAKF. Instead, it is computed using the total population count and the EAKF-adjusted state variables as$S_{ij}=N{-E}_{ij}{-R}_{ij}$. This guarantees population mass balance and is feasible under the assumption of non-reinfection among individuals. Additionally, it prevents the variable $S_{ij}$ from exceeding the total population, which aligns with the assumption of a constant population.

To properly balance the influence of the observational data in the assimilation process, it is crucial to estimate its error, which is typically unknown. Here, we estimated the Observational Error Variance of the observational data as shown in **Eq. S17,** similar to prior works [7–9]:

1. ${OEV}_{t}^{l}=max(5,\frac{{{(O}_{t}^{l})}^{2}}{100})$

${OEV}_{t}^{l}$ Observational Error Variance of location $l$ at time $t$

$O_{t}^{l}$ average cases in location $l$ in the week before time $t$

Repeated filter adjustments tend to decrease model ensemble variance reducing the impact of new observations in subsequent estimates. This may lead to divergence, in which the filter ceases adjusting the model state [10]. To counter divergence, at each timestep we applied a multiplicative factor (1.01) to inflate the prior ensemble of the observed variable (daily reported infected $I^{r}$) and the estimated parameters (ascertainment rate $\alpha$ and transmission rate $\beta$). Additionally, we reinitialized the values of the estimated parameters for a fraction (2%) of the ensemble members every 7 days. This reinitialization enables the system to periodically readjust estimations whenever the ensemble variance begins to shrink, thus preventing divergence.

Supplementary Note 4**:**

**Ascertainment rate initialization and lower bound**

Canada, the United States, and Mexico showed significant differences in healthcare and testing capacities during the pandemic. Mexico had particularly low testing rates [11,12], suggesting that only severe cases were assayed due to limited availability of test kits. During 2020, the United States experienced the highest number of cases, as well as relatively high nation-wide ascertainment rates, as shown in prior inference studies [13,14]. For Canada, the estimate of national testing rates or the ascertainment rate is lacking; however, Ontario, the most populous province in Canada, accounting for ~37% of the population, initially faced challenges in ramping up its testing capacity, but its centralized resource strategy was able to increase test capacity during 2020 [15].

Given these differences among the three countries, we assigned country-specific initial ranges for the ascertainment rate $\alpha$. For the US we used SARS-CoV-2 seroprevalence estimates and cumulative reported cases to estimate the value of the ascertainment rate. Specifically we used infection-induced seroprevalence from blood samples collected in US during July 2020 (3.5%) [16] to derive an initial estimate of $\alpha$ in the US, this formula is shown in **Eq. S18**:

1. $\alpha_{US}^{0}= median(\frac{\sum{cases}_{j}}{{IIS}_{US} \times{pop}_{j}})$

$\alpha_{US}^{0}$ Initial ascertainment rate for the US

$\sum{cases}_{j}$ Cumulative reported cases of state j up to July 2020

${IIS}_{US}$ Infection-induced seroprevalence in the United States during July 2020 (3.5%) [16]

${pop}_{j}$ Population of state j

Based on this formula, we set the initial prior distribution mean to 0.25 for $\alpha$ in US.

Estimated seroprevalence for SARS-CoV-2 in Canada was 1.9% in July 2020 [17], and in Mexico, it was 3.5% in February 2020[18]. Applying **Eq. S18** to these estimates produces very low initial $\alpha$ values (0.026 for Canada and 0 for Mexico), due to low numbers of reported cases and despite a measurable fraction of the population showing infection-induced seropositivity at the time. These issues may reflect low testing rates, as witnessed in Mexico [11,12], and early issues establishing testing facilities, as seen in Canada [15]. Therefore, we adjusted **Eq. S18** for Canada and Mexico and modeled the initial ascertainment rate distribution as a shifted exponential distribution. Specifically:

1. $\alpha_{CA,MX}^{0}\sim Exp\left( \lambda\right)+ \alpha_{min}$

$\alpha_{CA,MX}^{0}$ Initial ascertainment rate distribution for Canada and Mexico

$\lambda$ Exponential distribution rate parameter (0.022)

$\alpha_{min}$ Ascertainment rate initial lower bound (0.025)

The lower bound of $\alpha$ was set to 0.025 corresponding to 5 reported cases per 200 infections. For Canada and US, this bound increased linearly by 0.5% at each day, or: $\alpha_{t+1}^{min}= \alpha_{t}^{min}*1.005$. This increase was imposed to reflect the efforts of the two countries to increase the detection capabilities of local and national health systems [15,19–21]. Conversely, Mexico implemented a sentinel surveillance system [22] in which only the hospitalized cases and 10% of the mild cases were tested [23]. Focusing testing efforts on symptomatic individuals is a cost-effective strategy, but it also greatly increases the proportion of unreported cases, resulting in lower ascertainment rates. For this reason, the lower $\alpha$ bound of Mexico did not increase over time in our model.

Supplementary Note 5**:**

**Exceptions to the national trends in the estimated parameters**

The estimated values shown in **Figure 3-5** and **Supplementary Table 4** tend to follow very strong national trends, but there are some exceptions. Some Canadian territories and provinces, such as Northwest Territories, Nunavut, Prince Edward Island, and Yukon, exhibited relatively high transmission rates $\beta$ during the estimation period. However, it's important to note that their combined population represents less than 0.8% of the Canadian population and they reported extremely low daily case counts (i.e., <10). These factors contributed to increased stochasticity in the model for these locations. With such a low signal-to-noise ratio, the system may be less reliable at estimating parameter values.

In the US, notable exceptions to national trends are found in Alabama, North Dakota, Connecticut, and Rhode Island (see **Supplementary Figure 3**). Due to a backlog in reporting, Alabama added 4,007 cases data relative to test performed from October 2020 to January 2021 on March 15th 2021 [24], which is also the third timepoint selected to show the estimated values in this work. This event led the SEIR-EAKF system to adjust $\beta$ to high values (1.62). During the fall of 2020, North Dakota experienced a significant surge in cases. Following the outbreak, the estimated susceptible population dropped to approximately 30%, and by March 2021, $\beta$ value estimates became extremely broad. When transmission value estimates exceeded the bound values of 0.2 and 4, they were resampled across the current ensemble spread. When the spread is very large, the ensemble's average values will tend towards the midpoint between the upper and lower bounds (2.1). This could explain the exceptionally high estimate for North Dakota on March 15, 2021 (1.61). Connecticut and Rhode Island are neighboring states with interconnected epidemiological dynamics, showing a similar pandemic trajectory (see **Supplementary Figure 3**). Both states experienced an initial outbreak in fall 2020, followed by a larger outbreak during the winter of 2020–2021. The relatively high $\beta$ values estimated on March 15, 2021 (1.2 for Connecticut and 1.6 for Rhode Island), can be attributed to the fact that the spring 2021 outbreak, which began in March, did not originate from a very low case count as observed in other states. Moreover, March 15, 2021, the date of the estimate, coincided with a rapid rise in cases. The SEIR-EAKF system likely responded to this trend shift by estimating higher infection rates compared to the national average.

As discussed in the main text, in Mexico $\beta$ generally decreased gradually over time; a notable exception was Distrito Federal (Mexico City, See **Figure 3**), the very densely populated capital of Mexico. The relatively high $\beta$ value at the end of the estimation in March 2021 (1.6) was not unexpected in this location. High population density allows for more contact opportunities, increasing the rate of transmission and the basic reproduction number as shown in previous analysis [14].

The hyperparameter $R_{t}$ (time-varying basic reproductive number) calculated by **Eq. 11**, is proportional to the values of $\beta$ and $\alpha$. For this reason, the $R_{t}$ of the locations with exceptionally high $\beta$ (described above) showed high basic reproductive number $R_{t}$.

Supplementary Note 6**:**

**SEIR-EAKF comparison with GLEAM platform**

In this study we developed a new model which employs a novel commuting matrix derived from national census data and surveys to simulate worker movements across North America (Canada, US, and Mexico), focusing primarily on daily work commutes while incorporating additional random movement in proportion to the commuting flows. Additionally, the model integrates the SEIR compartmental model with the Ensemble Adjustment Kalman Filter (EAKF) to adjust system state variables and parameters based on case data, distinguishing it from multi-national models such as the GLEAM platform [25–27]. Those platforms estimate the movement of individuals among various arbitrary subpopulations located around major transportation hubs and leverages commuting and air travel data. In contrast, our approach utilizes state, province, and territory boundaries to define locations and is not informed by air travel data. Instead of adjusting the estimation, the GLEAM platform uses case data and other datasets to conduct a calibration run aimed at identifying the optimal set of parameters that best fit the real data in its estimations [28].

The role of asymptomatic individuals has been shown to be central for the spread of viruses such as SARS-CoV-2 and influenza, with most transmissions occurring with exposure times exceeding an hour [29]. Therefore, it is reasonable to center a model system around work-related commuting; infectious and asymptomatic individuals share the same space with coworkers for several hours, increasing their probability of infection; subsequently, each worker returns home to their resident locations, further increasing the probability of spreading disease to their families. Conversely, the GLEAM platform focuses on global disease spread, showing that the global spatiotemporal patterns of disease spreading are mainly determined by the airline network [30]. Additionally, the inclusion of air travel data enables GLEAM to capture global phenomena such as the external introduction or reintroduction of. In contrast, the SEIR-EAKF model developed in this work is more sensitive to local changes in trends, as it leverages case data to adjust estimations. The importance of modeling each introduction event is particularly relevant at the beginning of an epidemic. This significance decreases once the epidemic in a region is primarily driven by local transmission dynamics. Our model accommodates the external introduction of new infections through stochastic integration rather than relying on international flight data. However, the inclusion of global air travel data could improve system estimation, particularly in the initial weeks following the introduction of virus and for epidemics less prevalent than COVID-19. These differences make the two modeling approaches particularly effective at capturing different aspects and phases of epidemics. Therefore, combining these and other approaches into a single model or multi-model ensemble in the future might improve the capacity to estimate and predict disease parameters.

Supplementary Figure 1*:* ***a)*** *Total reported daily cases per country divided by the population. The dotted vertical lines indicates the three timepoints selected to show estimates in the main text. Source:* [*https://health.google.com/covid-19/open-data/data-sources*](https://health.google.com/covid-19/open-data/data-sources) ***b)*** *Percentage daily “change in workplace visitors” (i.e. individuals that commute to work) compared to baseline. The baseline is the median value, for the corresponding day of the week, during the 5-week period from January 3^rd^ to February 6^th^ 2020. The light curves represent each location, the thicker curves represent the average rate of the locations of each country. Source:* [*https://www.google.com/covid19/mobility/*](https://www.google.com/covid19/mobility/)

***
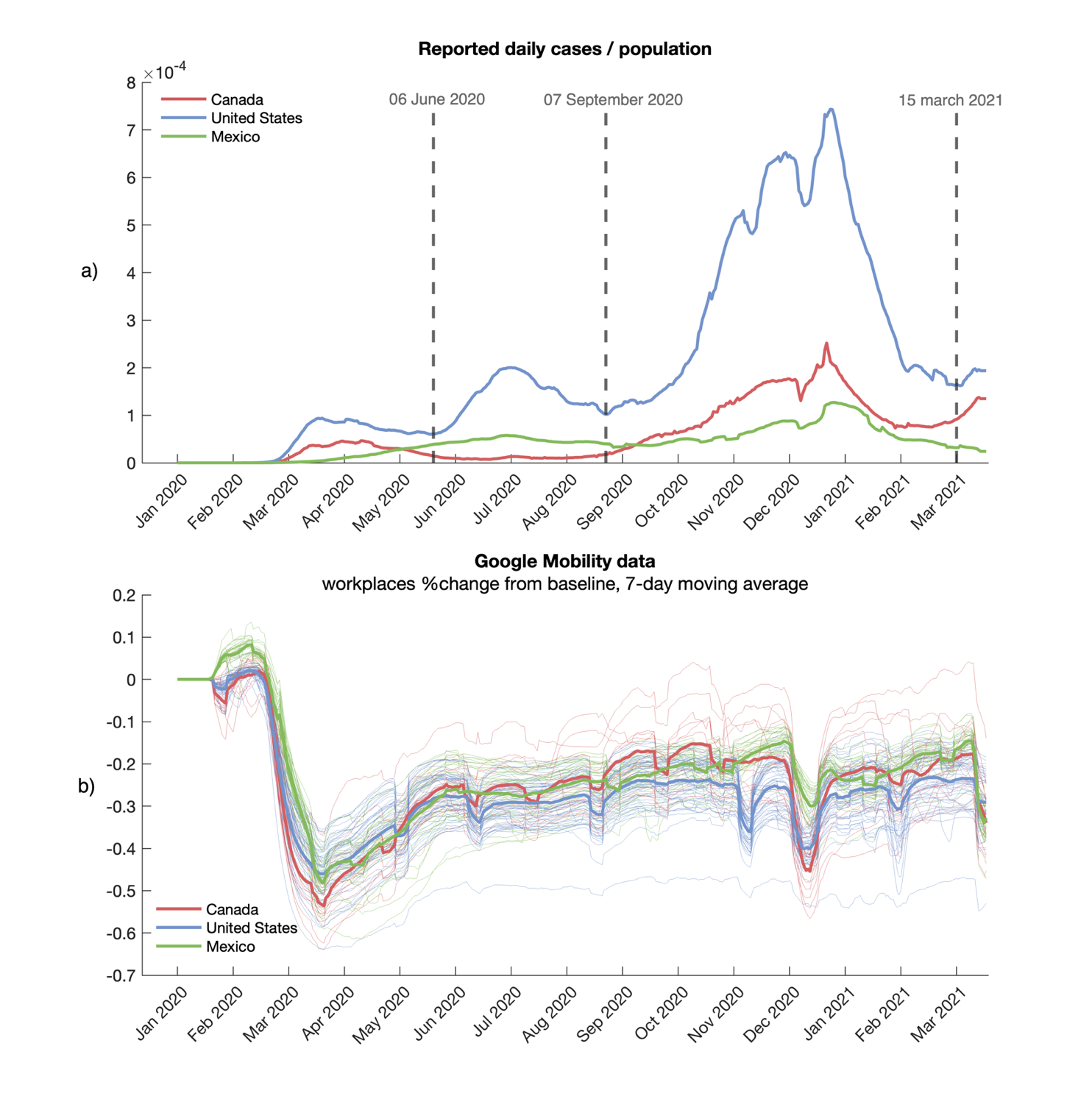
***


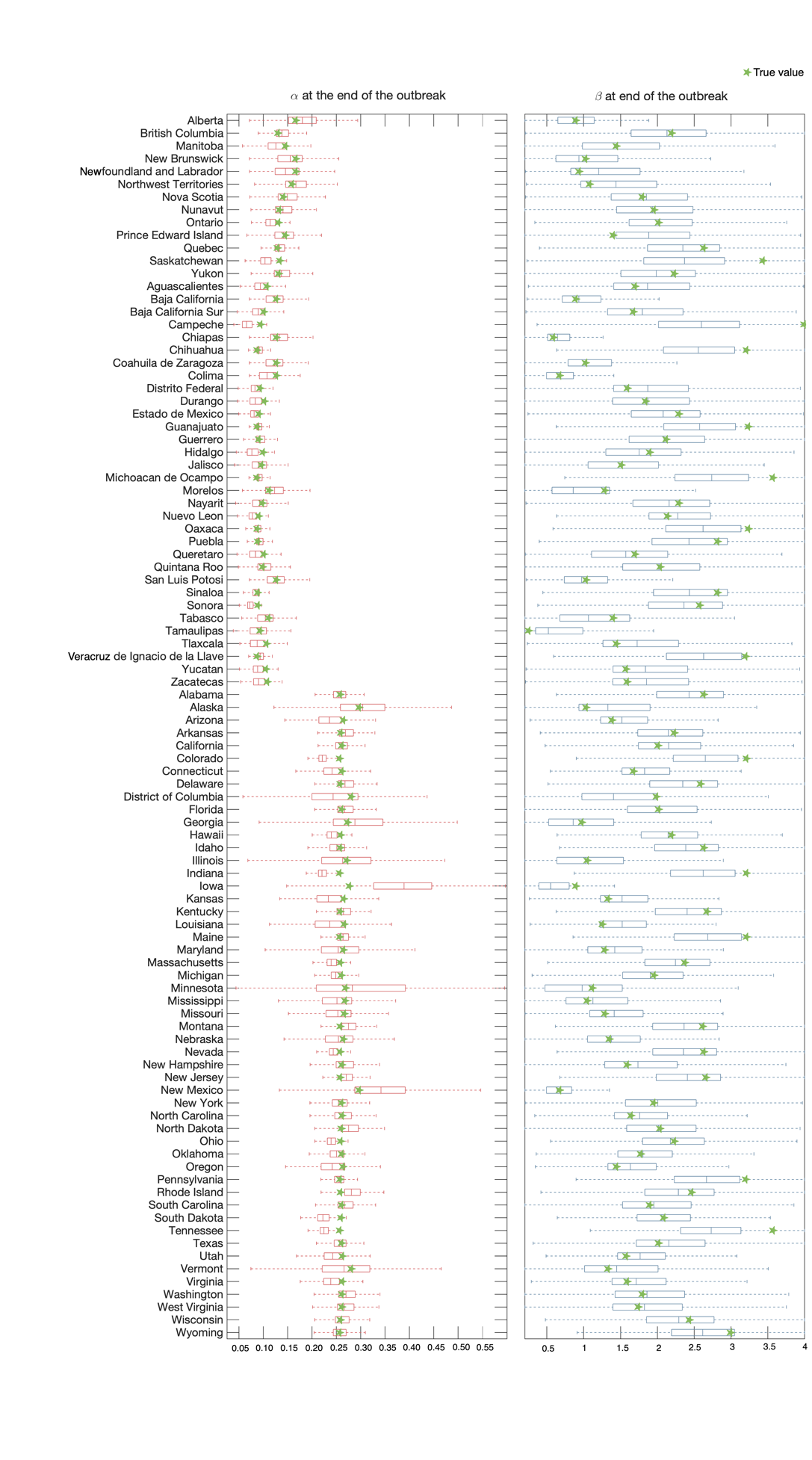
Supplementary Figure 2*:* *Boxplot distributions of parameter estimates and synthetic true values. Each box represent the interquartile range (IQR) with the median line inside the box. Whiskers extend to the minimum and maximum values within 1.5 times the IQR; outliers are not shown.*

Supplementary Figure 3*:* *Three US locations deviate from national trends in estimated parameters. Model fitting (i.e. estimated observed variable over the 7-day smoothed daily new reported cases, the model input), three state variables illustrating disease progression (the susceptible population, cumulative reported infectious and cumulative unreported infectious), and the parameters* $\alpha$*,* $\beta$ *and* $R_{t}$ *are shown for Alabama, North Dakota, Connecticut, and Rhode Island. The color shaded areas represent the 95% credible interval from the 300-member ensemble. The dotted vertical lines indicate the three timepoints of reference (June 6, 2020;* *September 7, 2020; March 15, 2021)*


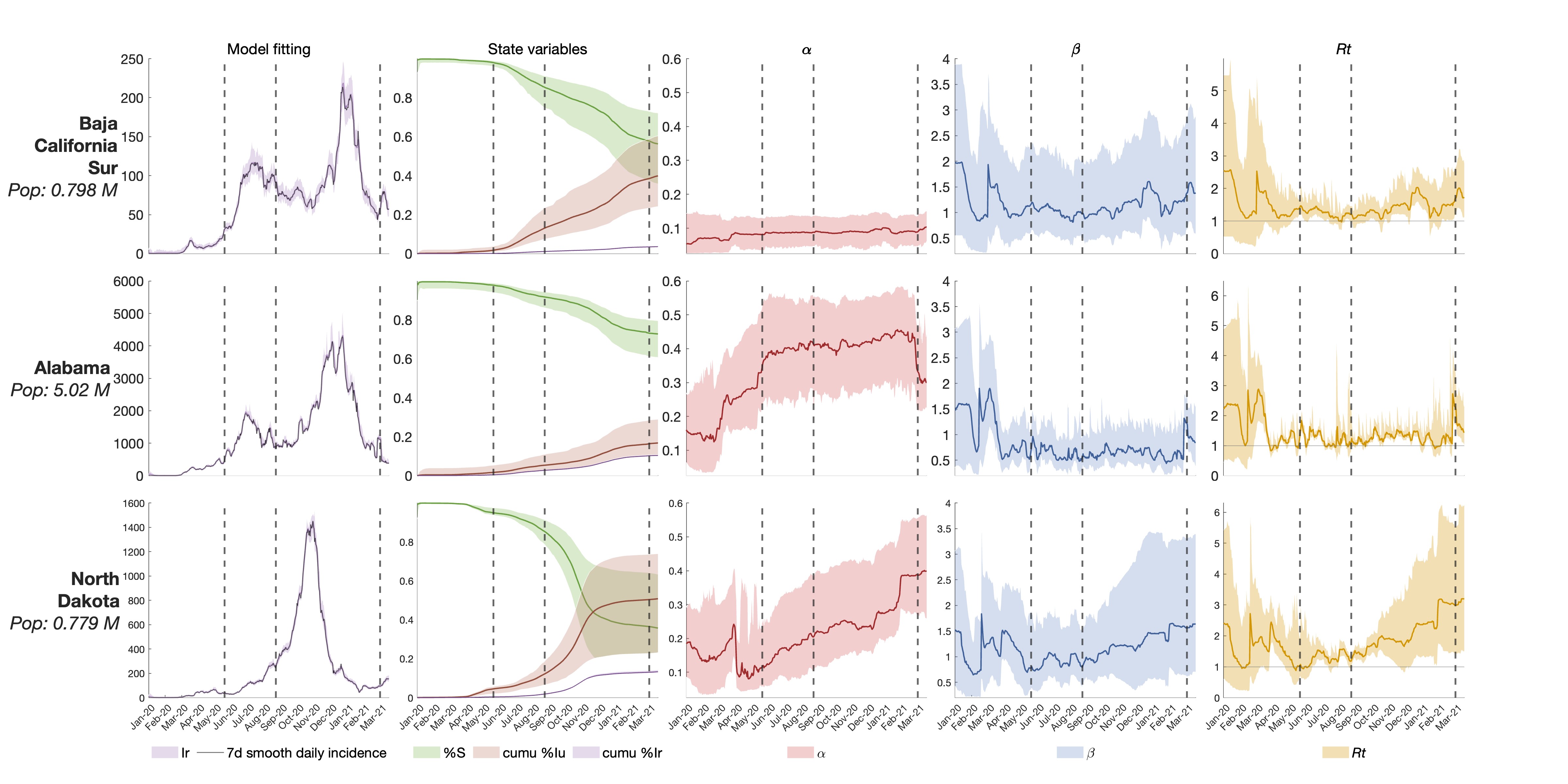

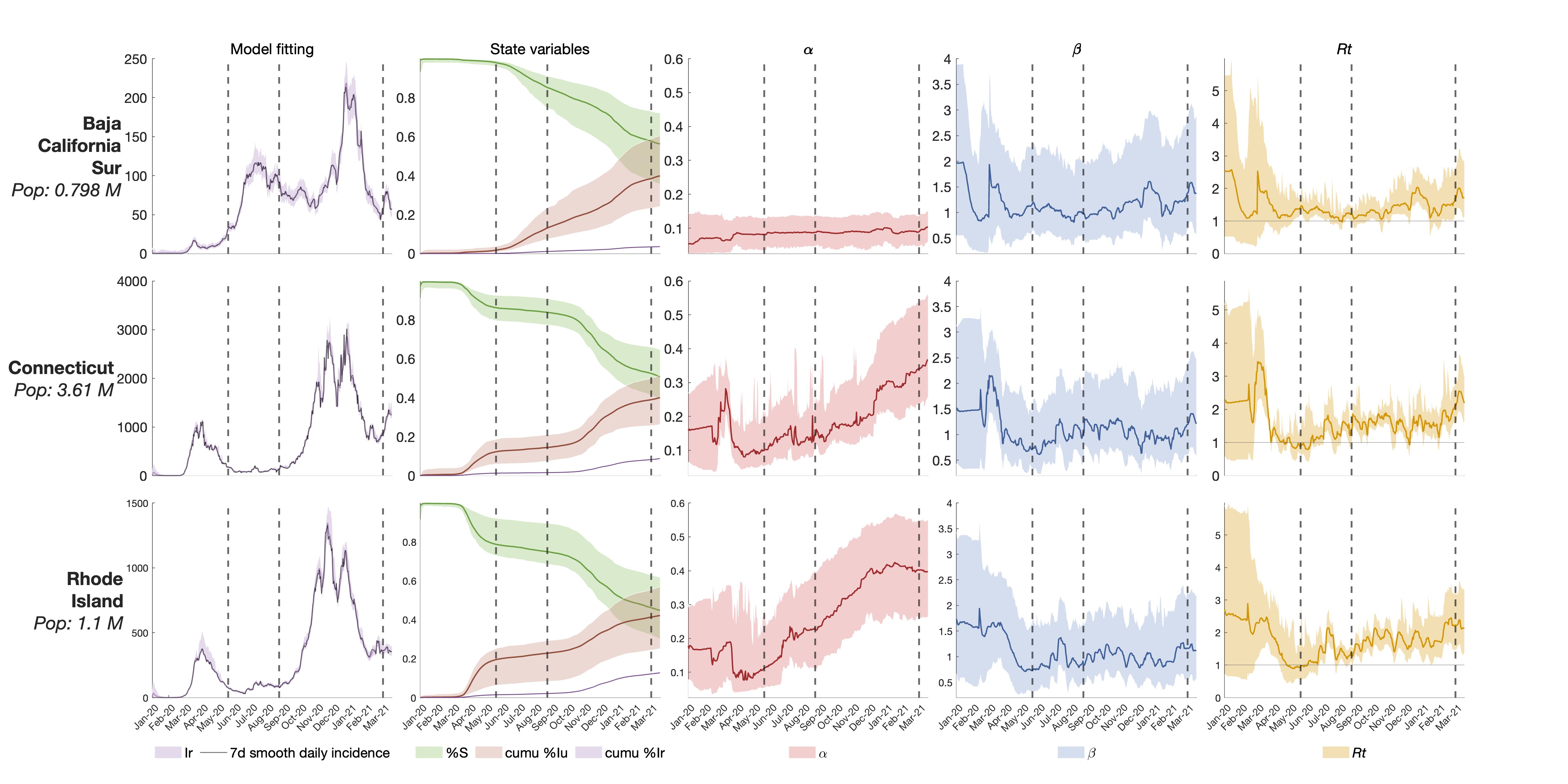

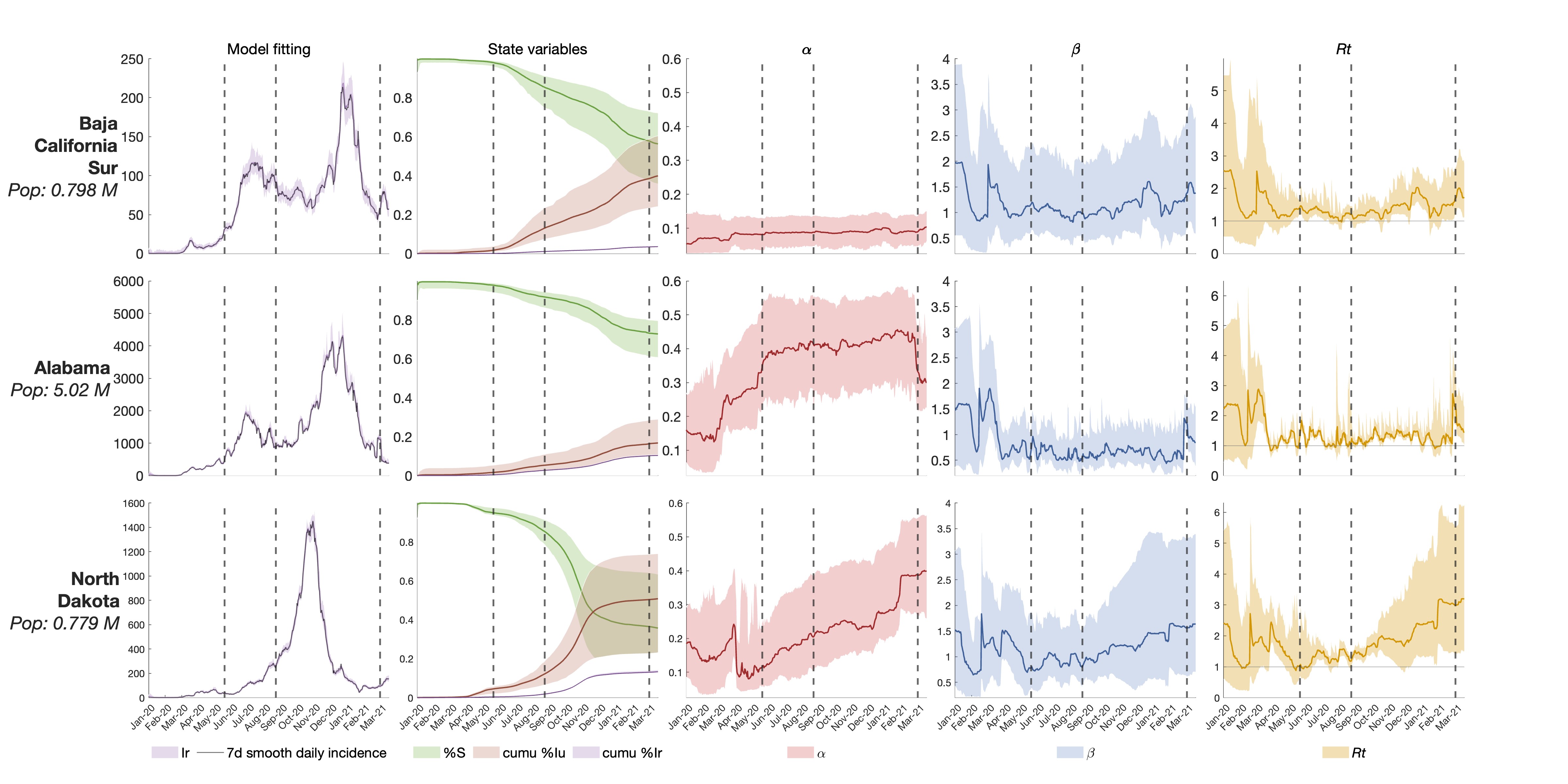


Supplementary Figure 4*:* *Irons and Raftery [31] estimated the infection undercount factor (total infections divided by total cases) for the US states on March 7^th^ 2021. The inverse of this factor is the ascertainment rate (fraction of documented infections) estimated in this work. The bar plots represent the 95% credible intervals and mean values.* *The intervals for Irons and Raftery and this study overlap for most states, indicating consistency across estimates; however, the distributions of the ascertainment rates estimated in this work are generally broader compared to the inverse of the infection undercount factor. In a few states, the infection undercount factor appears unrealistically high.*

*
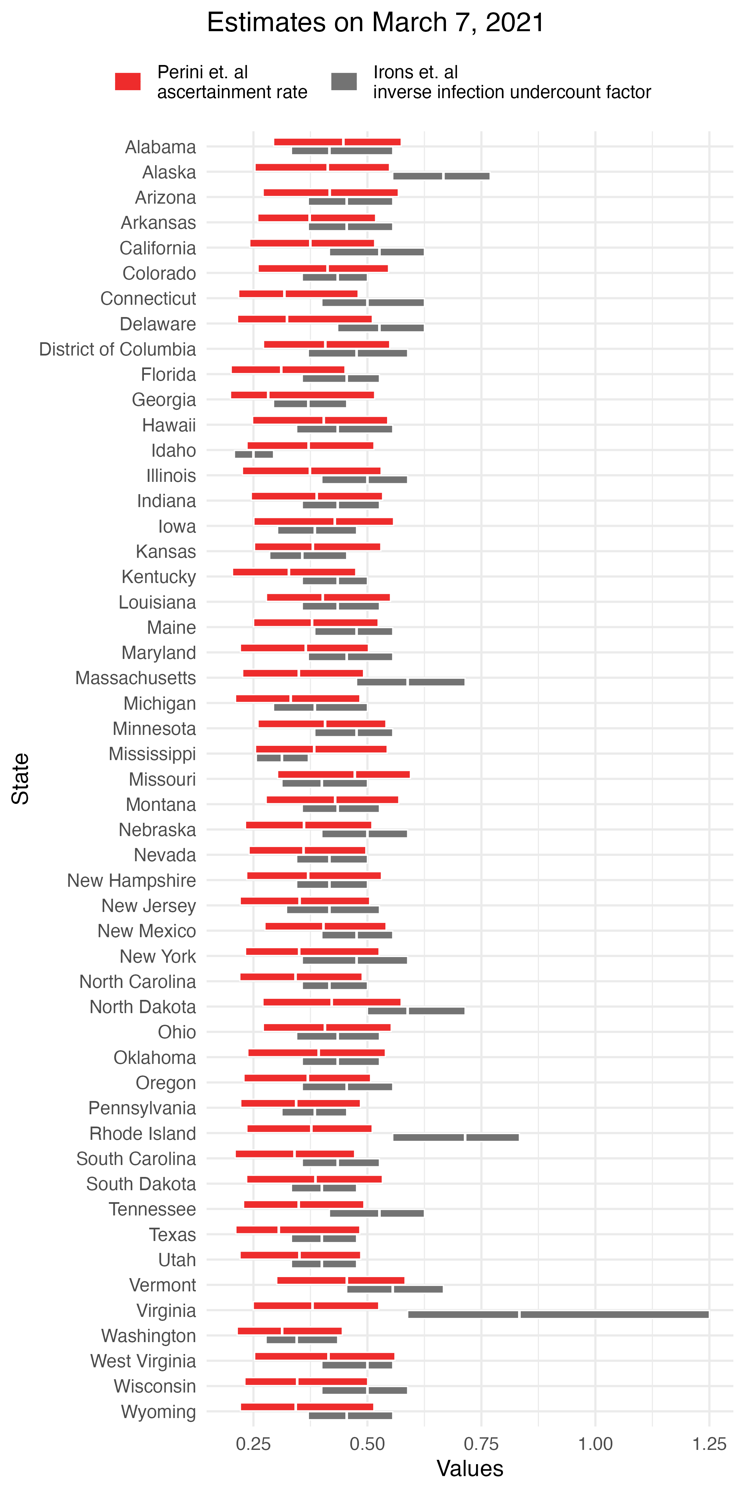
*

Supplementary Table 3*: Population-weighted average of the mean parameter estimates (ascertainment rate* $\alpha$ *and transmission rate* $\beta$*) and hyperparameter estimates (time-varying basic reproductive number* $R_{t}$*) for the three countries at three selected timepoints. The intensity of cell colors in the table corresponds to their values: higher values are represented by more intense colors*

| **Location** | **Jun. 6th 2020** | **Sep. 7th 2020** | **Mar. 15th 2021** |
| --- | --- | --- | --- |
|  | **Ascertainment rate** $\boldsymbol{\alpha}$ | | |
| Canada | 0.17 | 0.18 | 0.26 |
| United States | 0.26 | 0.31 | 0.36 |
| Mexico | 0.10 | 0.11 | 0.13 |
|  | **Transmission rate** $\boldsymbol{\beta}$ | | |
| Canada | 0.72 | 0.95 | 0.84 |
| United States | 0.72 | 0.64 | 0.85 |
| Mexico | 1.03 | 0.86 | 0.85 |
|  | **Time-varying basic reproduction number** $\boldsymbol{R}_{\boldsymbol{t}}$ | | |
| Canada | 0.99 | 1.32 | 1.31 |
| United States | 1.11 | 1.04 | 1.49 |
| Mexico | 1.22 | 1.04 | 1.07 |

Supplementary Table 4*:* *Mean parameter estimates* *(ascertainment rate* $\alpha$ *and transmission rate* $\beta$*) and hyperparameter estimates (basic reproductive number* $R_{t}$*) for all the locations at three selected timepoints. The intensity of cell colors in the table corresponds to their values: higher values are represented by more intense colors.*

| **Location** | | **June 6th 2020** | | | **September 7th 2020** | | | **March 15th 2021** | | |
| --- | --- | --- | --- | --- | --- | --- | --- | --- | --- | --- |
|  |  | $\boldsymbol{\alpha}$ | $\boldsymbol{\beta}$ | $\boldsymbol{R}_{\boldsymbol{t}}$ | $\boldsymbol{\alpha}$ | $\boldsymbol{\alpha}$ | $\boldsymbol{\beta}$ | $\boldsymbol{R}_{\boldsymbol{t}}$ | $\boldsymbol{\beta}$ | $\boldsymbol{\alpha}$ |
| CA | Alberta | 0.144 | 0.866 | 1.188 | 0.198 | 0.889 | 1.310 | 0.245 | 0.940 | 1.511 |
| CA | British Columbia | 0.133 | 0.739 | 0.966 | 0.168 | 0.881 | 1.258 | 0.245 | 0.725 | 1.154 |
| CA | Manitoba | 0.127 | 1.244 | 1.636 | 0.159 | 0.657 | 0.981 | 0.261 | 0.841 | 1.377 |
| CA | New Brunswick | 0.098 | 1.320 | 1.744 | 0.117 | 1.114 | 1.517 | 0.238 | 0.627 | 1.038 |
| CA | Newfoundland and Labrador | 0.111 | 1.145 | 1.623 | 0.152 | 1.090 | 1.640 | 0.254 | 1.014 | 1.790 |
| CA | Northwest Territories | 0.093 | 1.706 | 2.250 | 0.138 | 1.793 | 2.562 | 0.257 | 1.161 | 1.964 |
| CA | Nova Scotia | 0.096 | 0.792 | 1.105 | 0.117 | 1.000 | 1.371 | 0.241 | 0.607 | 1.052 |
| CA | Nunavut | 0.090 | 1.909 | 2.536 | 0.121 | 1.912 | 2.746 | 0.245 | 1.238 | 2.188 |
| CA | Ontario | 0.106 | 0.763 | 0.937 | 0.125 | 1.012 | 1.348 | 0.247 | 0.852 | 1.357 |
| CA | Prince Edward Island | 0.092 | 1.322 | 1.682 | 0.139 | 1.411 | 1.982 | 0.264 | 0.891 | 1.557 |
| CA | Quebec | 0.115 | 0.527 | 0.673 | 0.162 | 1.089 | 1.549 | 0.256 | 0.759 | 1.221 |
| CA | Saskatchewan | 0.145 | 0.810 | 1.177 | 0.175 | 0.975 | 1.432 | 0.231 | 0.709 | 1.101 |
| CA | Yukon | 0.094 | 1.486 | 1.928 | 0.121 | 1.480 | 2.038 | 0.244 | 1.015 | 1.725 |
| US | Alabama | 0.324 | 0.648 | 1.139 | 0.415 | 0.544 | 1.089 | 0.337 | 1.230 | 2.297 |
| US | Alaska | 0.253 | 0.938 | 1.488 | 0.408 | 0.578 | 1.112 | 0.433 | 0.644 | 1.290 |
| US | Arizona | 0.325 | 0.911 | 1.599 | 0.361 | 0.521 | 0.935 | 0.457 | 0.529 | 1.138 |
| US | Arkansas | 0.348 | 0.767 | 1.374 | 0.286 | 0.699 | 1.164 | 0.288 | 0.865 | 1.449 |
| US | California | 0.229 | 0.705 | 1.053 | 0.352 | 0.490 | 0.880 | 0.419 | 0.611 | 1.211 |
| US | Colorado | 0.349 | 0.499 | 0.934 | 0.393 | 0.534 | 1.003 | 0.426 | 0.567 | 1.108 |
| US | Connecticut | 0.182 | 0.584 | 0.816 | 0.144 | 1.016 | 1.379 | 0.340 | 1.196 | 2.116 |
| US | Delaware | 0.115 | 0.656 | 0.832 | 0.297 | 0.705 | 1.190 | 0.363 | 0.825 | 1.521 |
| US | District of Columbia (Washington DC) | 0.288 | 0.595 | 0.968 | 0.360 | 0.616 | 1.125 | 0.459 | 0.581 | 1.178 |
| US | Florida | 0.264 | 0.865 | 1.395 | 0.335 | 0.576 | 1.003 | 0.354 | 0.772 | 1.392 |
| US | Georgia | 0.219 | 0.775 | 1.168 | 0.277 | 0.622 | 1.007 | 0.312 | 1.005 | 1.720 |
| US | Hawaii | 0.225 | 1.069 | 1.701 | 0.321 | 0.519 | 0.901 | 0.420 | 0.628 | 1.248 |
| US | Idaho | 0.279 | 0.711 | 1.166 | 0.350 | 0.548 | 0.994 | 0.389 | 0.773 | 1.475 |
| US | Illinois | 0.268 | 0.516 | 0.818 | 0.309 | 0.681 | 1.147 | 0.387 | 0.824 | 1.563 |
| US | Indiana | 0.254 | 0.588 | 0.905 | 0.366 | 0.539 | 0.980 | 0.404 | 0.711 | 1.372 |
| US | Iowa | 0.257 | 0.649 | 1.032 | 0.350 | 0.553 | 0.981 | 0.435 | 0.760 | 1.503 |
| US | Kansas | 0.275 | 0.586 | 0.948 | 0.259 | 0.635 | 1.002 | 0.346 | 0.746 | 1.338 |
| US | Kentucky | 0.202 | 0.840 | 1.265 | 0.297 | 0.590 | 0.995 | 0.377 | 0.697 | 1.302 |
| US | Louisiana | 0.292 | 0.636 | 1.056 | 0.306 | 0.658 | 1.120 | 0.430 | 0.651 | 1.297 |
| US | Maine | 0.223 | 0.619 | 0.927 | 0.315 | 0.553 | 0.958 | 0.364 | 0.680 | 1.264 |
| US | Maryland | 0.230 | 0.535 | 0.793 | 0.266 | 0.739 | 1.203 | 0.355 | 0.782 | 1.430 |
| US | Massachusetts | 0.231 | 0.559 | 0.840 | 0.297 | 0.590 | 0.989 | 0.359 | 0.812 | 1.493 |
| US | Michigan | 0.292 | 0.769 | 1.509 | 0.344 | 0.641 | 1.157 | 0.349 | 1.020 | 1.875 |
| US | Minnesota | 0.341 | 0.477 | 0.850 | 0.438 | 0.455 | 0.910 | 0.448 | 0.711 | 1.446 |
| US | Mississippi | 0.286 | 0.651 | 1.067 | 0.407 | 0.484 | 0.932 | 0.449 | 0.635 | 1.279 |
| US | Missouri | 0.301 | 0.659 | 1.095 | 0.358 | 0.581 | 1.063 | 0.363 | 0.594 | 1.150 |
| US | Montana | 0.232 | 0.865 | 1.343 | 0.377 | 0.561 | 1.034 | 0.414 | 0.805 | 1.593 |
| US | Nebraska | 0.239 | 0.558 | 0.844 | 0.362 | 0.606 | 1.098 | 0.439 | 0.690 | 1.370 |
| US | Nevada | 0.253 | 0.905 | 1.456 | 0.347 | 0.498 | 0.893 | 0.435 | 0.621 | 1.221 |
| US | New Hampshire | 0.212 | 0.582 | 0.876 | 0.224 | 0.895 | 1.408 | 0.345 | 0.763 | 1.389 |
| US | New Jersey | 0.270 | 0.507 | 0.838 | 0.245 | 0.820 | 1.303 | 0.336 | 1.102 | 1.967 |
| US | New Mexico | 0.169 | 0.889 | 1.226 | 0.288 | 0.569 | 0.938 | 0.374 | 0.727 | 1.348 |
| US | New York | 0.182 | 0.594 | 0.806 | 0.307 | 0.663 | 1.124 | 0.364 | 0.740 | 1.358 |
| US | North Carolina | 0.299 | 0.745 | 1.251 | 0.368 | 0.500 | 0.919 | 0.373 | 0.661 | 1.231 |
| US | North Dakota | 0.235 | 0.733 | 1.126 | 0.204 | 0.796 | 1.177 | 0.388 | 1.562 | 2.997 |
| US | Ohio | 0.336 | 0.506 | 0.891 | 0.399 | 0.545 | 1.028 | 0.393 | 0.688 | 1.311 |
| US | Oklahoma | 0.295 | 0.689 | 1.150 | 0.441 | 0.579 | 1.150 | 0.439 | 0.591 | 1.181 |
| US | Oregon | 0.290 | 0.983 | 1.671 | 0.319 | 0.569 | 0.988 | 0.370 | 0.576 | 1.068 |
| US | Pennsylvania | 0.225 | 0.562 | 0.838 | 0.268 | 0.687 | 1.116 | 0.365 | 0.806 | 1.484 |
| US | Rhode Island | 0.210 | 0.610 | 0.890 | 0.232 | 0.914 | 1.423 | 0.402 | 1.165 | 2.228 |
| US | South Carolina | 0.243 | 0.914 | 1.433 | 0.311 | 0.620 | 1.054 | 0.369 | 0.733 | 1.365 |
| US | South Dakota | 0.407 | 0.547 | 1.049 | 0.430 | 0.542 | 1.042 | 0.414 | 0.833 | 1.627 |
| US | Tennessee | 0.307 | 0.654 | 1.092 | 0.286 | 0.642 | 1.069 | 0.420 | 0.856 | 1.694 |
| US | Texas | 0.338 | 0.688 | 1.209 | 0.365 | 0.504 | 0.910 | 0.308 | 0.869 | 1.501 |
| US | Utah | 0.277 | 0.886 | 1.457 | 0.317 | 0.657 | 1.131 | 0.389 | 0.764 | 1.438 |
| US | Vermont | 0.273 | 1.092 | 1.843 | 0.325 | 0.751 | 1.512 | 0.449 | 0.534 | 1.094 |
| US | Virginia | 0.278 | 0.538 | 0.863 | 0.266 | 0.668 | 1.088 | 0.383 | 0.746 | 1.412 |
| US | Washington | 0.217 | 0.799 | 1.191 | 0.272 | 0.617 | 1.006 | 0.309 | 0.716 | 1.244 |
| US | West Virginia | 0.275 | 0.662 | 1.084 | 0.424 | 0.588 | 1.143 | 0.424 | 0.720 | 1.433 |
| US | Wisconsin | 0.304 | 0.510 | 0.862 | 0.322 | 0.747 | 1.312 | 0.377 | 0.835 | 1.551 |
| US | Wyoming | 0.159 | 0.754 | 1.068 | 0.295 | 0.682 | 1.163 | 0.342 | 0.749 | 1.351 |
| MX | Aguascalientes | 0.087 | 1.136 | 1.375 | 0.084 | 0.823 | 0.990 | 0.106 | 0.853 | 1.073 |
| MX | Baja California | 0.108 | 0.911 | 1.140 | 0.114 | 0.879 | 1.128 | 0.136 | 0.697 | 0.911 |
| MX | Baja California Sur | 0.105 | 1.149 | 1.446 | 0.088 | 0.983 | 1.203 | 0.089 | 1.358 | 1.674 |
| MX | Campeche | 0.076 | 1.088 | 1.297 | 0.101 | 0.720 | 0.889 | 0.118 | 0.856 | 1.107 |
| MX | Chiapas | 0.083 | 0.825 | 0.978 | 0.098 | 0.810 | 1.038 | 0.133 | 0.687 | 0.938 |
| MX | Chihuahua | 0.088 | 0.815 | 0.984 | 0.083 | 0.868 | 1.045 | 0.104 | 0.934 | 1.186 |
| MX | Coahuila | 0.103 | 1.137 | 1.420 | 0.100 | 0.750 | 0.922 | 0.120 | 0.755 | 0.971 |
| MX | Colima | 0.068 | 1.155 | 1.348 | 0.085 | 0.758 | 0.912 | 0.102 | 0.851 | 1.071 |
| MX | Distrito Federal (Mexico City) | 0.107 | 0.858 | 1.062 | 0.111 | 0.947 | 1.197 | 0.134 | 1.497 | 1.991 |
| MX | Durango | 0.073 | 1.329 | 1.576 | 0.076 | 0.849 | 1.009 | 0.104 | 0.962 | 1.215 |
| MX | Estado de México | 0.095 | 0.919 | 1.129 | 0.093 | 0.853 | 1.046 | 0.102 | 0.788 | 0.987 |
| MX | Guanajuato | 0.106 | 1.230 | 1.547 | 0.098 | 0.842 | 1.047 | 0.112 | 0.850 | 1.083 |
| MX | Guerrero | 0.090 | 0.914 | 1.095 | 0.141 | 0.873 | 1.172 | 0.152 | 0.727 | 0.989 |
| MX | Hidalgo | 0.094 | 0.832 | 1.012 | 0.108 | 0.741 | 0.934 | 0.126 | 0.739 | 0.971 |
| MX | Jalisco | 0.101 | 1.234 | 1.533 | 0.104 | 0.867 | 1.095 | 0.107 | 0.715 | 0.906 |
| MX | Michoacán | 0.102 | 1.049 | 1.305 | 0.107 | 0.802 | 1.008 | 0.110 | 0.761 | 0.978 |
| MX | Morelos | 0.090 | 0.844 | 1.016 | 0.098 | 0.749 | 0.927 | 0.111 | 0.876 | 1.105 |
| MX | Nayarit | 0.078 | 1.061 | 1.250 | 0.087 | 0.828 | 1.006 | 0.117 | 0.775 | 1.006 |
| MX | Nuevo León | 0.117 | 1.221 | 1.585 | 0.116 | 0.826 | 1.053 | 0.144 | 0.735 | 0.971 |
| MX | Oaxaca | 0.094 | 1.125 | 1.388 | 0.090 | 0.934 | 1.156 | 0.111 | 0.804 | 1.020 |
| MX | Puebla | 0.099 | 1.012 | 1.266 | 0.098 | 0.788 | 0.967 | 0.089 | 0.834 | 1.019 |
| MX | Querétaro | 0.115 | 0.909 | 1.152 | 0.102 | 0.763 | 0.937 | 0.125 | 0.923 | 1.191 |
| MX | Quintana Roo | 0.090 | 0.909 | 1.093 | 0.101 | 0.767 | 0.942 | 0.128 | 0.894 | 1.185 |
| MX | San Luis Potosí | 0.103 | 1.243 | 1.582 | 0.104 | 0.751 | 0.934 | 0.123 | 0.850 | 1.103 |
| MX | Sinaloa | 0.081 | 1.012 | 1.200 | 0.090 | 0.840 | 1.020 | 0.104 | 0.891 | 1.122 |
| MX | Sonora | 0.090 | 0.966 | 1.166 | 0.110 | 0.827 | 1.038 | 0.127 | 0.930 | 1.219 |
| MX | Tabasco | 0.106 | 1.073 | 1.353 | 0.115 | 0.757 | 0.954 | 0.132 | 0.924 | 1.220 |
| MX | Tamaulipas | 0.095 | 1.022 | 1.257 | 0.114 | 0.918 | 1.176 | 0.135 | 0.816 | 1.082 |
| MX | Tlaxcala | 0.076 | 0.972 | 1.147 | 0.093 | 0.736 | 0.899 | 0.109 | 0.919 | 1.192 |
| MX | Veracruz | 0.081 | 0.892 | 1.063 | 0.076 | 0.856 | 1.024 | 0.098 | 0.764 | 0.961 |
| MX | Yucatán | 0.090 | 0.944 | 1.152 | 0.104 | 0.883 | 1.110 | 0.132 | 0.847 | 1.119 |
| MX | Zacatecas | 0.073 | 1.184 | 1.405 | 0.097 | 0.803 | 0.996 | 0.126 | 0.766 | 1.003 |

# References

1. Government of Canada SC. Commuting Flow from Geography of Residence to Geography of Work - Census Metropolitan Areas and Census Agglomerations: Main Mode of Commuting (10), Commuting Duration (6) and Sex (3) for the Employed Labour Force Aged 15 Years and Over Having a Usual Place of Work, in Private Households, 2016 Census - 25% Sample Data [Internet]. 2017 [cited 2024 Apr 3]. Available from: https://www12.statcan.gc.ca/census-recensement/2016/dp-pd/dt-td/Rp-eng.cfm?TABID=4&LANG=E&A=R&APATH=3&DETAIL=0&DIM=0&FL=A&FREE=0&GC=0&GL=-1&GID=1354564&GK=0&GRP=1&O=D&PID=111333&PRID=10&PTYPE=109445&S=0&SHOWALL=0&SUB=0&Temporal=2017&THEME=125&VID=0&VNAMEE=&VNAMEF=%20(2017)&D1=0&D2=0&D3=0&D4=0&D5=0&D6=0

2. Statistics Canada. Number of vehicles travelling between Canada and the United States [Internet]. Government of Canada; [cited 2023 Dec 22]. Available from: https://www150.statcan.gc.ca/t1/tbl1/en/tv.action?pid=2410000201

3. US Census Bureau. 2011-2015 5-Year ACS Commuting Flows [Internet]. [cited 2023 Dec 22]. Available from: https://www.census.gov/data/tables/2015/demo/metro-micro/commuting-flows-2015.html

4. INEGI. Intercensal Survey 2015 [Internet]. [cited 2023 Dec 22]. Available from: https://en.www.inegi.org.mx/programas/intercensal/2015/#Microdatos

5. Guerra E, Benitez JP, Caudillo C, et al. Transportation and Land Use across US and Mexican Urban Areas. [cited 2024 Jun 25]; . Available from: https://rosap.ntl.bts.gov/view/dot/54764

6. Diekmann O, Heesterbeek JAP, Roberts MG. The construction of next-generation matrices for compartmental epidemic models. J R Soc Interface [Internet]. **2010** [cited 2024 Jan 23]; 7(47):873–885. Available from: https://royalsocietypublishing.org/doi/10.1098/rsif.2009.0386

7. Yang W, Karspeck A, Shaman J. Comparison of Filtering Methods for the Modeling and Retrospective Forecasting of Influenza Epidemics. Ferguson NM, editor. PLoS Comput Biol [Internet]. **2014** [cited 2024 Jan 23]; 10(4):e1003583. Available from: https://dx.plos.org/10.1371/journal.pcbi.1003583

8. Pei S, Kandula S, Yang W, Shaman J. Forecasting the spatial transmission of influenza in the United States. Proc Natl Acad Sci [Internet]. **2018** [cited 2024 Jan 23]; 115(11):2752–2757. Available from: https://pnas.org/doi/full/10.1073/pnas.1708856115

9. Li R, Pei S, Chen B, et al. Substantial undocumented infection facilitates the rapid dissemination of novel coronavirus (SARS-CoV-2). Science [Internet]. **2020** [cited 2023 Dec 22]; 368(6490):489–493. Available from: https://www.science.org/doi/10.1126/science.abb3221

10. Anderson JL. An Ensemble Adjustment Kalman Filter for Data Assimilation. Mon Weather Rev [Internet]. **2001** [cited 2023 Dec 22]; 129(12):2884–2903. Available from: http://journals.ametsoc.org/doi/10.1175/1520-0493(2001)129<2884:AEAKFF>2.0.CO;2

11. Dahal S, Banda JM, Bento AI, Mizumoto K, Chowell G. Characterizing all-cause excess mortality patterns during COVID-19 pandemic in Mexico. BMC Infect Dis [Internet]. **2021** [cited 2024 Mar 20]; 21(1):432. Available from: https://bmcinfectdis.biomedcentral.com/articles/10.1186/s12879-021-06122-7

12. Hasell J, Mathieu E, Beltekian D, et al. A cross-country database of COVID-19 testing. Sci Data [Internet]. **2020** [cited 2024 Mar 20]; 7(1):345. Available from: https://www.nature.com/articles/s41597-020-00688-8

13. Pei S, Yamana TK, Kandula S, Galanti M, Shaman J. Burden and characteristics of COVID-19 in the United States during 2020. Nature [Internet]. **2021** [cited 2024 Jan 19]; 598(7880):338–341. Available from: https://www.nature.com/articles/s41586-021-03914-4

14. Sen Pei, Kandula S, Shaman J. Differential effects of intervention timing on COVID-19 spread in the United States. Sci Adv [Internet]. **2020** [cited 2023 Dec 22]; 6(49):eabd6370. Available from: https://www.science.org/doi/10.1126/sciadv.abd6370

15. Lee MH-Y, Xu G, Cheng F, Khalid AF. Testing surge capacity—A Canadian COVID-19 experience, Ontario’s surge capacity for the first wave. Health Policy [Internet]. **2021** [cited 2024 Mar 26]; 125(10):1291–1296. Available from: https://linkinghub.elsevier.com/retrieve/pii/S0168851021002025

16. Jones JM, Stone M, Sulaeman H, et al. Estimated US Infection- and Vaccine-Induced SARS-CoV-2 Seroprevalence Based on Blood Donations, July 2020-May 2021. JAMA [Internet]. **2021** [cited 2024 Jan 12]; 326(14):1400. Available from: https://jamanetwork.com/journals/jama/fullarticle/2784013

17. Tang X, Sharma A, Pasic M, et al. Assessment of SARS-CoV-2 Seropositivity During the First and Second Viral Waves in 2020 and 2021 Among Canadian Adults. JAMA Netw Open [Internet]. **2022** [cited 2024 Dec 2]; 5(2):e2146798. Available from: https://jamanetwork.com/journals/jamanetworkopen/fullarticle/2789086

18. Muñoz-Medina JE, Grajales-Muñiz C, Salas-Lais AG, et al. SARS-CoV-2 IgG Antibodies Seroprevalence and Sera Neutralizing Activity in MEXICO: A National Cross-Sectional Study during 2020. Microorganisms [Internet]. **2021** [cited 2024 Dec 2]; 9(4):850. Available from: https://www.mdpi.com/2076-2607/9/4/850

19. The COVID Tracking Project [Internet]. COVID Track. Proj. [cited 2024 Feb 26]. Available from: https://covidtracking.com/data/national

20. Canada PHA of. COVID-19 daily epidemiology update: Testing and variants [Internet]. aem. 2020 [cited 2024 Feb 26]. Available from: https://www.canada.ca/en.html

21. Mathieu E, Ritchie H, Rodés-Guirao L, et al. Coronavirus Pandemic (COVID-19). Our World Data [Internet]. **2020** [cited 2024 Feb 26]; . Available from: https://ourworldindata.org/coronavirus-testing

22. Dirección General de Epidemiología. Lineamiento estandarizado para la vigilancia epidemiológica y por laboratorio de enfermedad por 2019-nCoV. 2020 [Internet]. Available from: https://www.gob.mx/salud/documentos/lineamiento-estandarizado-para-la-vigilancia-epidemiologica-y-por-laboratorio-de-la-enfermedad-respiratoria-viral

23. De La Cruz-Hernández SI. Another Vision of the Situation of the COVID-19 Pandemic in Mexico During 2020. Disaster Med Public Health Prep [Internet]. **2022** [cited 2024 Apr 23]; 16(6):2296–2298. Available from: https://www.cambridge.org/core/product/identifier/S1935789321003402/type/journal_article

24. Leada Gore. Alabama adds 4,556 COVID cases, 4,007 from backlog dating back to 2020 [Internet]. al. 2021 [cited 2024 Apr 25]. Available from: https://www.al.com/news/2021/03/alabama-adds-4556-covid-cases-4007-from-backlog-dating-back-to-2020.html

25. Balcan D, Gonçalves B, Hu H, Ramasco JJ, Colizza V, Vespignani A. Modeling the spatial spread of infectious diseases: The GLobal Epidemic and Mobility computational model. J Comput Sci [Internet]. **2010** [cited 2024 Jan 22]; 1(3):132–145. Available from: https://linkinghub.elsevier.com/retrieve/pii/S1877750310000438

26. Pastore-Piontti A, Zhang Q, Gomes MFC, et al. Real-Time Assessment of the International Spreading Risk Associated with the 2014 West African Ebola Outbreak. In: Chowell G, Hyman JM, editors. Math Stat Model Emerg Re-Emerg Infect Dis [Internet]. Cham: Springer International Publishing; 2016 [cited 2024 Jan 23]. p. 39–56. Available from: http://link.springer.com/10.1007/978-3-319-40413-4_4

27. Zhang Q, Sun K, Chinazzi M, et al. Spread of Zika virus in the Americas. Proc Natl Acad Sci [Internet]. **2017** [cited 2024 Jan 23]; 114(22). Available from: https://pnas.org/doi/full/10.1073/pnas.1620161114

28. Davis JT, Chinazzi M, Perra N, et al. Cryptic transmission of SARS-CoV-2 and the first COVID-19 wave. Nature [Internet]. Nature Publishing Group; **2021** [cited 2024 May 1]; 600(7887):127–132. Available from: https://www.nature.com/articles/s41586-021-04130-w

29. Ferretti L, Wymant C, Petrie J, et al. Digital measurement of SARS-CoV-2 transmission risk from 7 million contacts. Nature [Internet]. Nature Publishing Group; **2024** [cited 2024 Mar 29]; 626(7997):145–150. Available from: https://www.nature.com/articles/s41586-023-06952-2

30. Balcan D, Colizza V, Gonçalves B, Hu H, Ramasco JJ, Vespignani A. Multiscale mobility networks and the spatial spreading of infectious diseases. Proc Natl Acad Sci [Internet]. Proceedings of the National Academy of Sciences; **2009** [cited 2024 Mar 29]; 106(51):21484–21489. Available from: https://www.pnas.org/doi/10.1073/pnas.0906910106

31. Irons NJ, Raftery AE. Estimating SARS-CoV-2 infections from deaths, confirmed cases, tests, and random surveys. Proc Natl Acad Sci [Internet]. **2021** [cited 2024 Dec 2]; 118(31):e2103272118. Available from: https://pnas.org/doi/full/10.1073/pnas.2103272118
